# Detection of Sleep Apnea Using Wearable AI: Systematic Review and Meta-Analysis

**DOI:** 10.2196/58187

**Published:** 2024-09-10

**Authors:** Alaa Abd-alrazaq, Hania Aslam, Rawan AlSaad, Mohammed Alsahli, Arfan Ahmed, Rafat Damseh, Sarah Aziz, Javaid Sheikh

**Affiliations:** 1 AI Center for Precision Health Weill Cornell Medicine-Qatar Qatar Foundation Doha Qatar; 2 Health Informatics Department, College of Health Science, Riyadh, Saudi Electronic university Riyadh Saudi Arabia; 3 Department of Computer Science and Software Engineering, United Arab Emirates University Al Ain Abu Dhabi United Arab Emirates

**Keywords:** sleep apnea, hypopnea, artificial intelligence, wearable devices, machine learning, systematic review, mobile phone

## Abstract

**Background:**

Early detection of sleep apnea, the health condition where airflow either ceases or decreases episodically during sleep, is crucial to initiate timely interventions and avoid complications. Wearable artificial intelligence (AI), the integration of AI algorithms into wearable devices to collect and analyze data to offer various functionalities and insights, can efficiently detect sleep apnea due to its convenience, accessibility, affordability, objectivity, and real-time monitoring capabilities, thereby addressing the limitations of traditional approaches such as polysomnography.

**Objective:**

The objective of this systematic review was to examine the effectiveness of wearable AI in detecting sleep apnea, its type, and its severity.

**Methods:**

Our search was conducted in 6 electronic databases. This review included English research articles evaluating wearable AI’s performance in identifying sleep apnea, distinguishing its type, and gauging its severity. Two researchers independently conducted study selection, extracted data, and assessed the risk of bias using an adapted Quality Assessment of Studies of Diagnostic Accuracy-Revised tool. We used both narrative and statistical techniques for evidence synthesis.

**Results:**

Among 615 studies, 38 (6.2%) met the eligibility criteria for this review. The pooled mean accuracy, sensitivity, and specificity of wearable AI in detecting apnea events in respiration (apnea and nonapnea events) were 0.893, 0.793, and 0.947, respectively. The pooled mean accuracy of wearable AI in differentiating types of apnea events in respiration (normal, obstructive sleep apnea, central sleep apnea, mixed apnea, and hypopnea) was 0.815. The pooled mean accuracy, sensitivity, and specificity of wearable AI in detecting sleep apnea were 0.869, 0.938, and 0.752, respectively. The pooled mean accuracy of wearable AI in identifying the severity level of sleep apnea (normal, mild, moderate, and severe) and estimating the severity score (Apnea-Hypopnea Index) was 0.651 and 0.877, respectively. Subgroup analyses found different moderators of wearable AI performance for different outcomes, such as the type of algorithm, type of data, type of sleep apnea, and placement of wearable devices.

**Conclusions:**

Wearable AI shows potential in identifying and classifying sleep apnea, but its current performance is suboptimal for routine clinical use. We recommend concurrent use with traditional assessments until improved evidence supports its reliability. Certified commercial wearables are needed for effectively detecting sleep apnea, predicting its occurrence, and delivering proactive interventions. Researchers should conduct further studies on detecting central sleep apnea, prioritize deep learning algorithms, incorporate self-reported and nonwearable data, evaluate performance across different device placements, and provide detailed findings for effective meta-analyses.

## Introduction

### Background

Sleep apnea refers to a health condition where airflow either ceases or decreases episodically during sleep [1]. According to the American Academy of Sleep Medicine, sleep apnea is categorized as a sleep disorder wherein an individual experiences challenges pertaining to breathing when they are asleep [2]. Primarily, there are 3 kinds of sleep apnea. First, obstructive sleep apnea (OSA) is the consequence of issues with the operation of the upper respiratory tract and is considered a chronic breathing disorder associated with sleep [3]. By contrast, a condition where signals required to regulate breathing muscles are not generated or transmitted is referred to as central sleep apnea (CSA). Complex or mixed sleep apnea is a condition that involves a combination of both OSA and CSA [4]. It often begins as OSA and evolves into CSA [4].

According to global estimates, approximately 936 million adults aged between 30 and 69 years experience OSA [5]. A systematic review showed that the global prevalence of OSA is between 9% and 38% [6]. In the United States alone, the number of people struggling with sleep apnea may exceed 30 million, as per the American Medical Association [7]. Furthermore, studies showed that >80% of sleep apnea cases remain undiagnosed [7-10]. If not diagnosed and treated, sleep apnea may result in severe health issues, such as mood disorders [11-13], cardiovascular diseases [14,15], cognitive deterioration [16,17], increased risk of road accidents [18,19], and all-cause mortality [20,21]. Therefore, the timely detection of sleep apnea for prompt initiation of treatment is imperative.

Conventionally, polysomnography is a comprehensive diagnostic test used in the field of sleep medicine to evaluate and monitor various physiological parameters during sleep to help diagnose sleep disorders, such as sleep apnea [22]. Despite being considered the gold standard for diagnosing sleep apnea, it does have some disadvantages and limitations: (1) it is relatively expensive; (2) access to sleep laboratories may be limited, particularly in certain geographic areas; (3) it can be inconvenient for patients, as they must spend a full night in a sleep laboratory with numerous sensors and electrodes attached to their body; (4) the physiological parameters recorded using polysomnography may not fully reflect the individual’s typical sleep behavior due to a first-night effect in a sleep laboratory, where sleep patterns are different from those at home due to the novelty of the environment; and (5) it is a subjective process, as analyzing polysomnography data depends on sleep clinicians’ experience [22-24]. Hence, there is a dire need to develop and integrate automated technologies and tools that are more efficient and capable of addressing the challenges posed by the current system of diagnosing sleep apnea.

One of the promising solutions that have been used to address the limitations of polysomnography is wearable artificial intelligence (AI), which refers to the integration of AI algorithms and techniques into wearable devices (eg, smartwatches, fitness trackers, and smart glasses) to collect and analyze data (eg, heart rate [HR], respiration rate, and oxygen saturation) to offer various functionalities and insights. Sleep apnea can be efficiently detected with wearable AI due to its convenience, accessibility, affordability, objectivity, and real-time monitoring capabilities. Various types of wearable devices can be used for gathering biomarkers associated with sleep apnea: on-body devices (worn directly on the body or skin), near-body devices (worn close to the body but not touching the skin), in-body devices (implanted within the body), and electronic textiles (clothes with built-in technology). Wearable AI can be used for (1) detecting apnea events in respiration, (2) identifying the type of apnea events in respiration (hypopnea, OSA, CSA, and mixed), (3) detecting patients with sleep apnea, and (4) estimating the severity of sleep apnea.

### Research Problem and Aim

In the last decade, numerous investigations have been carried out to evaluate the effectiveness of wearable AI in detecting sleep apnea. Consolidating the results of these studies can contribute to forming more conclusive judgments regarding the effectiveness of wearable AI in detecting sleep apnea. Previous literature reviews attempted to summarize the evidence, but they were constrained by the following limitations. First, most previous reviews were literature reviews rather than systematic reviews [22-28]. Second, many reviews concentrated solely on OSA rather than considering all types of sleep apnea [22,23,25-29]. Third, some reviews focused on a specific type of data, such as HR variability [2,25] and electrocardiography [1,2,25], for sleep apnea detection. Fourth, main databases, such as Embase [1,2,22-29], ACM [1,2,22-29], IEEE [22-25,27-29], and Scopus [1,2,22-25,28], were not incorporated in the searches of previous reviews. Fifth, all prior reviews focused on the performance of various sensors rather than specifically addressing wearable devices [1,2,22-29]. Sixth, one of the reviews focused on non-AI tools for detecting sleep apnea [29]. Seventh, the risk of bias was not taken into account in most of the reviews [1,2,22-28]. Finally, none of these reviews used statistical techniques (eg, meta-analysis) to aggregate findings from previous studies [1,2,22-29]. Hence, this review aimed to bridge all these identified gaps with a focus on examining the performance of wearable AI when it comes to both the detection and prediction of sleep apnea, thereby making it the first of its kind in this field.

## Methods

### Overview

This review was undertaken and reported in line with the PRISMA-DTA (Preferred Reporting Items for Systematic Reviews and Meta-Analyses extension for Diagnostic Test Accuracy) guidelines [30]. Multimedia Appendix 1 provides this review’s PRISMA-DTA checklist. Its protocol has been registered with the PROSPERO (CRD42023495554).

### Search Strategy

On December 7, 2023, a comprehensive search was performed across the following electronic repositories: MEDLINE (via Ovid), Embase (via Ovid), ACM Digital Library, Scopus, IEEE Xplore, and Google Scholar. MEDLINE and Embase were chosen due to their reputation as authoritative sources for biomedical and health sciences literature. ACM Digital Library and IEEE Xplore were selected for their status as leading repositories for publications in computing, information technology, electrical engineering, and electronics. Scopus was included because of its comprehensive coverage of scientific literature across multiple disciplines, including health sciences, engineering, computer science, and social sciences. Google Scholar was incorporated, as it indexes scholarly literature from diverse sources and serves as a valuable supplementary tool for identifying relevant studies and gray literature. We set an autoalert to run the search query biweekly for 3 months, concluding on March 6, 2024. Because Google Scholar returned a massive number of results, this review assessed only the first 100 results (equivalent to 10 pages). To identify additional relevant studies, we examined the references cited in the studies already included (backward reference list checking) and studies that had cited the included studies (forward reference list checking).

Relevant literature reviews were assessed, and 2 experts holding doctoral degrees in digital health and health informatics were consulted to compile and collate search terms [31]. The final search query combined three categories of search terms: (1) terms related to AI (eg, “artificial intelligence,” “machine learning,” and “deep learning”), (2) terms associated with wearable devices (eg, “wearable,” “smartwatch,” and “smartband”), and (3) terms linked to sleep apnea (eg, “sleep apnea” and “sleep aponea”). The Boolean operators “OR” and “AND” were used to combine terms within the same category and across different categories, respectively. The specific search query used for searching each database is detailed in Multimedia Appendix 2 for reference.

### Study Eligibility Criteria

This review included studies that used AI algorithms to detect sleep apnea or predict its occurrence by leveraging data derived from wearable devices. The research articles deemed suitable for inclusion in this review were those that concentrated on individuals diagnosed with or suspected of having any type of sleep apnea. No limitations were imposed based on age, gender, or ethnicity. In addition, for inclusion in this review, studies were required to evaluate the performance of AI algorithms in detecting or predicting apnea events in respiration, identifying types of apnea events in respiration, detecting patients with sleep apnea, or estimating the severity of sleep apnea. The studies had to provide the confusion matrix or performance metrics (eg, accuracy, sensitivity, and specificity). Studies using AI solely for detecting sleep quality, sleep stages, or other sleep disorders or forecasting the outcomes of sleep apnea interventions were excluded. This review included studies that gathered data using, at a minimum, on-body wearable devices. Conversely, research papers exclusively relying on the following devices for data collection were not considered: nonwearable devices; handheld devices (eg, mobile phones); near-body wearable devices; in-body wearable devices; wearable devices physically connected to nonwearable devices; and wearable devices necessitating expert oversight, such as those demanding precise electrode placement. This review included only peer-reviewed journal articles, conference papers, and dissertations, without restrictions on study setting, study design, reference standard (ie, ground truth), year of publication, or country of study. However, papers not published in English were excluded from consideration. The decision to exclude studies not written in English was based on practical considerations related to resource constraints and the accessibility of non-English literature. While including studies in languages other than English may enhance the comprehensiveness of the review, it can also pose challenges in terms of language translation, interpretation, and the synthesis of findings. Furthermore, English is widely recognized as the dominant language of scholarly communication in many scientific disciplines, including health care and biomedical research. We have transparently acknowledged its implications for the review’s scope and findings in the *Limitations* section. We also excluded studies that fell into the categories of editorials, reviews, protocols, posters, conference abstracts, and research highlights. The decision to exclude these publication types was primarily guided by the need to maintain the focus and rigor of our review process. While editorials, reviews, and research highlights provide valuable insights into and perspectives on a topic, they typically do not present original research findings or empirical data that meet the objectives of our study. Similarly, protocols, posters, and conference abstracts often offer preliminary or incomplete results that may not undergo peer review or provide sufficient detail for a comprehensive analysis. This helps maintain the quality and reliability of the evidence synthesized in our review while minimizing the risk of bias introduced by including non–peer-reviewed or preliminary findings.

### Study Selection

The study selection process comprised 3 key steps. Initially, the EndNote (version X9; Clarivate) software was used to eliminate any duplicate papers from the initial pool. Subsequently, 2 reviewers assessed the titles and abstracts of the remaining studies, separately deciding on their inclusion. Finally, the reviewers independently scrutinized the full texts of the remaining articles. Any discrepancies were deliberated upon and resolved through discussion. The level of agreement between the reviewers was substantial, indicated by a κ score of 0.92 for the evaluation of titles and abstracts and 0.95 for the examination of full texts.

### Data Extraction

Initially, 5 studies were used to develop and test the data extraction form shown in Multimedia Appendix 3. Independently, 2 reviewers used Excel (Microsoft Corp) to extract metadata from the studies, participants’ characteristics, wearable devices’ specifications, and AI algorithms’ features. In addition to the previously mentioned extracted data, we collected the highest performance score for each metric, algorithm, and measured outcome. When studies provided raw data or confusion matrices, we calculated all possible performance metrics (eg, accuracy, specificity, and sensitivity). In case of the unavailability of such data, we attempted to obtain them by reaching out to the studies’ first and corresponding authors. Any discrepancies between the 2 reviewers were addressed through discussion between them.

### Risk of Bias and Applicability Appraisal

To evaluate the quality of the studies included in our review, we adapted the Quality Assessment of Studies of Diagnostic Accuracy-Revised (QUADAS-2) [32] tool to align with our review’s specific objectives. This adaptation involved substituting some of the original criteria, which were not applicable to our context, with more relevant criteria from the Prediction Model Risk of Bias Assessment Tool [33]. We modified the QUADAS-2 tool to encompass 4 main domains tailored to our review: “participants,” “index test” (focused on AI algorithms), “reference standard” (representing the ground truth), and “analysis.” For each domain, we developed 4 targeted questions aligned with our review’s objectives. In addition, our evaluation assessed the practical applicability of the results derived from the first 3 domains. To optimize our adapted tool, we initially tested it on 5 studies for fine-tuning purposes. The included studies were independently evaluated by 2 reviewers using the modified QUADAS-2 tool (Multimedia Appendix 4). Any differences in their assessments were discussed and resolved through consensus.

### Data Synthesis

We used both narrative and statistical techniques to synthesize the data extracted from the included studies. In our narrative synthesis, we used textual descriptions and tabulated summaries to elucidate the characteristics of the included studies, encompassing study metadata, wearable devices, and AI techniques. As for the statistical approach, a meta-analysis was carried out when at least 2 different studies presented enough data to perform the analysis. We conducted conventional meta-analyses for results associated with the following outcomes, given that they were extracted from different unique studies (ie, independent effect sizes): identification of types of apnea events in respiration, detection of patients with sleep apnea, and estimation of the severity of sleep apnea. Specifically, DerSimonian-Laird random-effects models [34] using the Freeman-Tukey double arcsine transformation [35,36] were performed to pool the extracted results. This method considers variations arising from sampling and accounts for heterogeneity in estimates. The analysis was carried out using the *meta* toolkit within R (version 4.2.2; The R Foundation) [37].

We also performed multilevel meta-analyses for results related to the detection of apnea events in respiration, as certain results originated from the same study (ie, dependent effect sizes) [34,38]. Multilevel meta-analyses were used to address this dependency in effect sizes, thereby minimizing the risk of type I errors. These analyses were carried out using the *metafor* toolkit within R (version 4.2.2) [35].

When applicable, subgroup meta-analyses were conducted to explore how different factors might influence the effectiveness of wearable AI [34,38]. These factors included AI algorithms, the type of algorithm (ie, machine learning [ML] vs deep learning), the number of participants, the type of sleep apnea, the status of the wearable device (ie, commercial vs noncommercial), the placement of the wearable device, data set size, data type, ground truth, and validation method. We considered differences in results between subgroups to be statistically significant if the statistical probability (*P* value) was <.05.

To assess how consistent the studies were in their findings (heterogeneity), we used 2 statistical tests. The first test is the Cochrane Q statistic, which indicates whether the observed differences in results could be due to chance alone. A *P* value <.05 indicates significant heterogeneity, meaning the results varied more than expected by chance. The second test is the *I*^2^ statistic, which quantifies the proportion of observed variability due to real differences between studies rather than differences by chance [35,39]. Heterogeneity was considered insignificant when *I*^2^ ranged from 0% to 40%, moderate when *I*^2^ fell within the 30% to 60% range, substantial when *I*^2^ ranged from 50% to 90%, or considerable when *I*^2^ extended from 75% to 100%.

## Results

### Search Results

**Figure 1:**
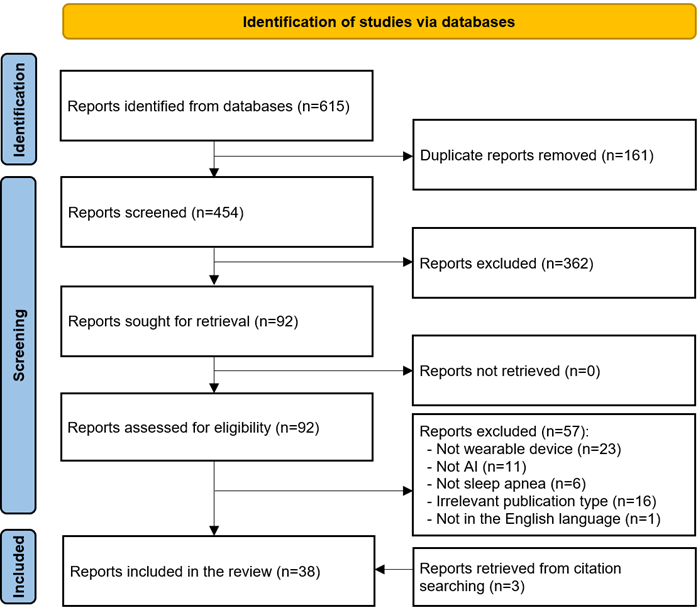
PRISMA (Preferred Reporting Items for Systematic Reviews and Meta-Analyses) flowchart of the study selection process. AI: artificial intelligence.

As depicted in Figure 1, a total of 615 citations were retrieved when the above-identified databases were searched. Of the retrieved citations, 161 (26.2%) duplicates were removed using EndNote X9, leaving 454 (73.8%) studies. Further, 362 (79.7%) studies were removed after screening the titles and abstracts of these 454 studies. After retrieving and reading the full text of all the remaining 92 (20.3%) studies, it was determined that 57 (62%) of these studies were ineligible for inclusion. The main reasons for exclusion were that they did not use wearable devices (23/92, 25%), did not use AI algorithms (11/92, 12%), did not focus on sleep apnea (6/92, 7%), were irrelevant publication types (16/92, 17%), or were not written in English (1/92, 1%). We identified 3 additional studies relevant to this review through backward reference list checking. In total, 38 studies were included in this review [40-77], and 27 (71%) of them were eligible for meta-analyses [40,41,45-49,52-55,57,58,61-64,66,68,69,71-77].

### Characteristics of the Included Studies

As displayed in Table 1, the number of studies has varied over the years, with the highest number reached in 2020 (11/38, 29%). While the included studies were conducted in 16 different countries, the studies were predominantly from the United States (9/38, 24%). Most of the studies were journal articles (29/38, 76%), but conference papers also made a substantial contribution (9/38, 24%). The average number of participants across studies was 155.9 (SD 374.9). The number of participants ranged from 4 to 2252. The mean age of participants was identified in 25 (66%) of the 38 included studies and ranged from 25.6 to 61.1 years, with an average of 47.3 (SD 9.3) years. Across 25 studies reporting the proportion of female participants, female participants constituted an average of 37.4% of the total participants, ranging from 12% to 65%. A total of 20 studies reported the BMI, which ranged from 22.1 to 38.7 kg/m^2^, with an average of 28.6 (SD 3.81) kg/m^2^. About two-thirds (25/38, 66%) of studies did not focus on a specific type of sleep apnea. The characteristics of each included study are listed in Multimedia Appendix 5.

**Table 1:** Characteristics of the included studies (N=38).

Features					Studies	References
**Year of publication, n (%)**						
		2023			8 (21)	[54,55,60,64,67,71,75,76]
		2022			9 (24)	[40,42,46,52,61,63,68,73,77]
		2021			4 (11)	[43,53,70,72]
		2020			11 (29)	[41,45,47,49,51,57-59,65,66,74]
		2019			1 (3)	[48]
		2018			3 (8)	[44,50,69]
		2014			1 (3)	[62]
		2013			1 (3)	[56]
**Country of publication, n (%)**						
			United States		9 (24)	[43,44,46,47,55-57,64,72]
			China		8 (21)	[42,63,67,68,70,71,75,76]
			South Korea		4 (11)	[45,51,73,77]
			Canada		2 (5)	[48,49]
			Italy		2 (5)	[40,60]
			Norway		2 (5)	[53,54]
			Taiwan		2 (5)	[41,69]
			Others (<2)		9 (24)	[50,52,58,59,61,62,65,66,74]
**Publication type, n (%)**						
				Conference paper	9 (24)	[43-45,48,50,51,59,62,76]
				Journal article	29 (76)	[40-42,46,47,49,52-58,60,61,63-75,77]
Number of participants, mean (SD; range)					155.9 (374.9; 4-2252)	[40-77]
**Age (y)**						
		Value, mean (SD; range)			47.3 (9.3; 25.6-61.1)	[40,41,43,45-49,53,54,57-62,64,66,67,69,71-73,75,77]
		Not reported, n (%)			13 (34)	[42,44,50-52,55,56,63,65,68,70,74,76]
**Female participants (%)**						
	Value, mean (SD; range)				37.4 (14.76; 12-65)	[40,41,43,46-49,52-54,57-62,64,66-69,71-73,75]
	Not reported, n (%)				13 (34)	[42,44,45,50,51,55,56,63,65,70,74,76,77]
**BMI (kg/m^2^)**						
			Value, mean (SD; range)		28.6 (3.813; 22.1-38.7)	[40,41,45,47-49,53,54,57,58,61,62,64,66,68,69,71-73,77]
			Not reported, n (%)		18 (47)	[42-44,46,50-52,55,56,59,60,63,65,67,70,74-76]
**Sleep apnea type, n (%)**						
			All		25 (66)	[41,43,45,46,48,49,51-55,57,59-63,66,67,69,70,73-75,77]
			Obstructive sleep apnea		12 (32)	[40,42,44,47,56,58,64,65,68,71,72,76]
			Central sleep apnea		1 (3)	[50]

### Features of Wearable Devices

Commercial wearable devices constituted the majority of wearable devices in the included studies (24/38, 63%; Table 2). The most mentioned wearable device in the included studies was the Belun Ring (3/38, 8%). Wearable devices are placed on various body parts, with the chest (16/38, 42%), wrist (11/38, 29%), and abdomen (9/38, 24%) being the most common locations. Wearable devices were worn for 1 full night (6-8 hours) in 29 studies (76%). The features of wearable devices in each included study are shown in Multimedia Appendix 6.

**Table 2:** Features of wearable devices (N=38).

Features			Studies, n (%)		References
**Status of the wearable device**					
	Commercial	24 (63)		[40,42-47,51,53,54,57,58,60-66,70,72,73,76,77]	
	Noncommercial	14 (37)		[41,48-50,52,55,56,59,67-69,71,74,75]	
**Name of the wearable device**					
	Belun Ring	3 (8)		[47,64,72]	
	Patch	2 (5)		[48,49]	
	T-REX TR100A	2 (5)		[73,77]	
	Others	17 (45)		[43-46,51,53,54,57,60-62,64-66,71,74,76]	
	Not reported	14 (37)		[41,42,50,52,55,56,58,59,63,67-70,75]	
**Placement of the wearable device**					
	Chest	16 (42)		[41,46,50,52-54,56,57,59-62,65,66,68,69]	
	Wrist	11 (29)		[40,42-45,51,54,58,63,70,76]	
	Abdomen	9 (24)		[41,53,54,57,65,69,73,74,77]	
	Finger	6 (16)		[47,53,64,67,69,72]	
	Neck	2 (5)		[48,49]	
	Nose	2 (5)		[53,75]	
	Face	1 (3)		[55]	
**Duration of wearing the wearable device**					
	1 full night	29 (76)		[41-43,45-49,53-56,58,60-69,71-73,75-77]	
	<1 full night	3 (8)		[50,52,59]	
	>1 full night	2 (5)		[44,51]	
	Not reported	3 (8)		[57,70,74]	

### Features of AI

Classification was the dominant problem-solving approach used in the included studies (38/38, 100%; Table 3). Various AI algorithms were used in the included studies, with convolutional neural networks (CNNs) being the most common (14/38, 37%). Among the 38 included studies, most studies (n=37, 97%) used AI to detect the current sleep apnea, whereas 3 (8%) studies used wearable AI to predict sleep apnea before its occurrence. The mean data set size reported in 28 (74%) studies was 60,554 (SD 133,059), with the range spanning from 12 to 561,480. Most studies (36/38, 95%) used closed-source data, while only 2 (5%) of 38 studies used open-source data. Data were gathered through wearable devices in all studies (38/38, 100%), via self-reported questionnaires in 3 (8%) studies, and using nonwearable devices (eg, smartphones) in 2 (5%) studies. Respiration data (eg, respiratory rate and respiratory efforts; 25/38, 66%) and HR data (eg, HR, HR variability, and interbeat interval; 21/38, 55%) were the most frequently used data for developing the models in the included studies. The number of features reported in 21 (55%) of the 38 studies ranged from 3 to 212, with an average of 44.3 (62.5). Most studies used polysomnography as the ground truth assessment method (26/38, 68%), followed by the wearable device (8/38, 21%) and the context of the experiment (eg, performing different patterns of breathing; 4/38, 11%). In 28 studies that reported the assessor of the ground truth, sleep technicians were the most common assessors (23/38, 61%), followed by sleep physicians (8/38, 21%). American Academy of Sleep Medicine guidelines were followed in 84% (32/38) of studies to assess the ground truth. Train-test split was the most common approach used in the included studies to validate the performance of AI models (20/38, 53%), followed by k-fold cross-validation (17/38, 45%). The included studies used wearable AI to detect apnea events in respiration (24/38, 63%) and patients with sleep apnea (15/38, 40%) and to identify the severity of sleep apnea (21/38, 55%) and types of apnea events in respiration (8/38, 21%). The features of AI in each included study are described in Multimedia Appendix 7.

**Table 3:** Features of artificial intelligence (N=38).

Features			Studies		References
**Problem-solving approaches, n (%)**					
	Classification	38 (100)		[40-77]	
	Regression	15 (40)		[45-49,54,56,58,62,64,70,71,76]	
**AI^a^ algorithms, n (%)**					
	Convolutional neural network	14 (37)		[48,49,53-55,57-60,63,64,68,73,75,77]	
	Random forest	10 (26)		[40,42,43,46,52-54,67,70,73]	
	Long short-term memory	9 (24)		[41,44,45,48,49,52-54,66]	
	Support vector machines	8 (21)		[43,52,53,56,62,67,69,73]	
	K-nearest neighbors	7 (18)		[42,51,52,54,61,67,70]	
	Artificial neural network	5 (13)		[47,51,65,72,74]	
	Multilayer perceptron	5 (13)		[40,50,53,54,73]	
	Naive Bayes	5 (13)		[41,42,51,52,70]	
	Decision trees	4 (11)		[42,43,52,70]	
	AdaBoost	3 (8)		[41,52,61]	
	Others (<3)	5 (13)		[52,67,71,73,76]	
**Aim of AI algorithms, n (%)**					
	Detection	37 (97)		[40-55,57-76]	
	Prediction	3 (8)		[44,56,65]	
**Data set size**					
	Value, mean (SD; range)	60,554 (133,059; 12-561,480)		[40,42-44,46-49,52-55,58-66,68,72-76]	
	Not reported, n (%)	10 (26)		[41,45,50,51,56,57,67,69-71]	
**Data sources, n (%)**					
	Closed source	36 (95)		[40-43,45-64,66-77]	
	Open source	2 (5)		[44,65]	
**Data types, n (%)**					
	Wearable device data	38 (100)		[40-77]	
	Self-reported data	3 (8)		[44,69,76]	
	Nonwearable device data	2 (5)		[69,76]	
**Data input to AI algorithms, n (%)**					
	Respiration data	25 (66)		[41,43,45,46,48-50,52-54,56-62,65,66,69,73-77]	
	Heart rate	21 (55)		[40,42,45,47,50-52,56,58,62-64,66-68,70-73,76,77]	
	Body movement	14 (37)		[40,44,45,47,51,52,58,60,62,64,66,71,72,76]	
	Oxygen saturation	13 (34)		[41,46,47,53,54,56,60,64,67,69,71,72,76]	
	Acoustic data	3 (8)		[56,60,76]	
	Others (<3)	10.5 (4)		[44,55,58,76]	
**Number of features**					
	Value, mean (SD; range)	44.33 (62.5; 3-212)		[40-43,45-50,52,56-58,61,64,65,69,70,72,73]	
	Not reported, n (%)	17 (45)		[44,51,53-55,59,60,62,63,66-68,71,73-77]	
**Ground truth assessment methods, n (%)**					
	Polysomnography	26 (68)		[41-43,45-49,55,58,61-73,75-77]	
	Wearable device	8 (21)		[40,44,51,53,54,56,60,74]	
	Context	4 (11)		[50,52,57,59]	
**Guidelines for ground truth assessment, n (%)**					
	American Academy of Sleep Medicine guidelines	32 (84)		[40-49,51,53-55,58,61-77]	
	Not reported	6 (16)		[50,52,56,57,59,60]	
**Assessors of ground truth, n (%)**					
	Sleep technician	23 (61)		[40-42,44-47,53-56,58,60-62,64-66,69,72,73,76,77]	
	Sleep physician	8 (21)		[42,57,63,64,68,71,72]	
	Not reported	10 (26)		[48-52,59,68,70,74,75]	
**Validation methods, n (%)**					
	Train-test split	20 (53)		[41,45,47,51,52,55,57-60,63-65,68,71-75,77]	
	K-fold cross-validation	17 (45)		[42-44,46,48-51,53,54,56,61,63,66,68,70,76]	
	Leave-one-out cross-validation	5 (13)		[40,54,60,62,69]	
	Not reported	1 (3)		[67]	
**Measured outcomes, n (%)**					
	Apnea events in respiration	24 (63)		[41,43-46,50-59,61,63,66-68,73-75,77]	
	Sleep apnea severity	21 (55)		[40-43,45-49,53,58,62,64,65,69-73,76,77]	
	Patients with sleep apnea	15 (40)		[40-44,46-48,53,58,63,64,69,71,72,76]	
	Type of apnea events	8 (21)		[41,46,53,57,58,60,66,75]	

^a^AI: artificial intelligence.

### Results of Risk-of-Bias Appraisal

**Figure 2:**
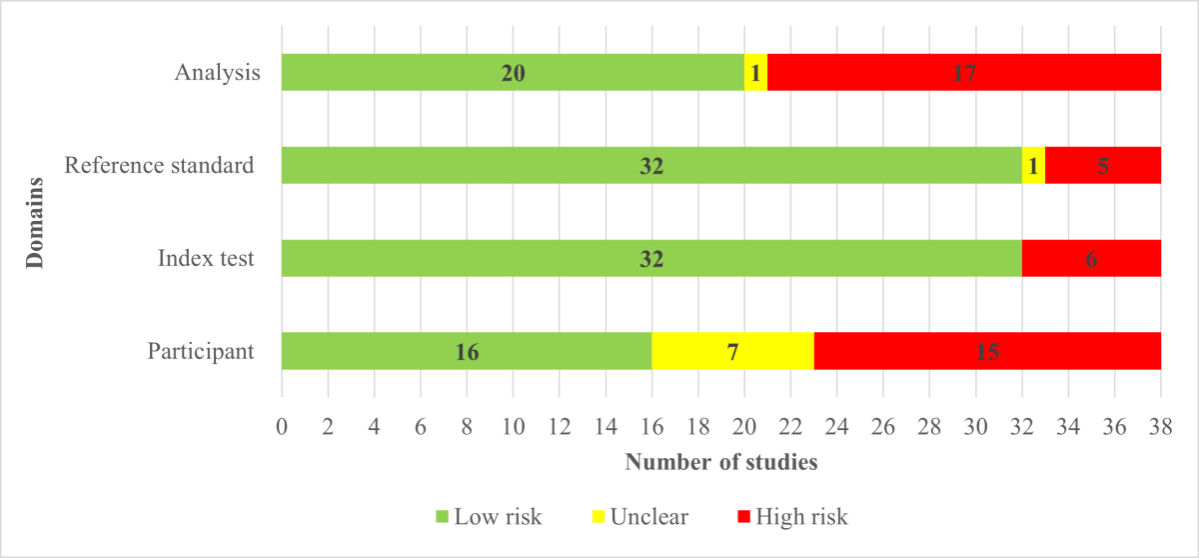
Results of the assessment of risk of bias in the included studies.

**Figure 3:**
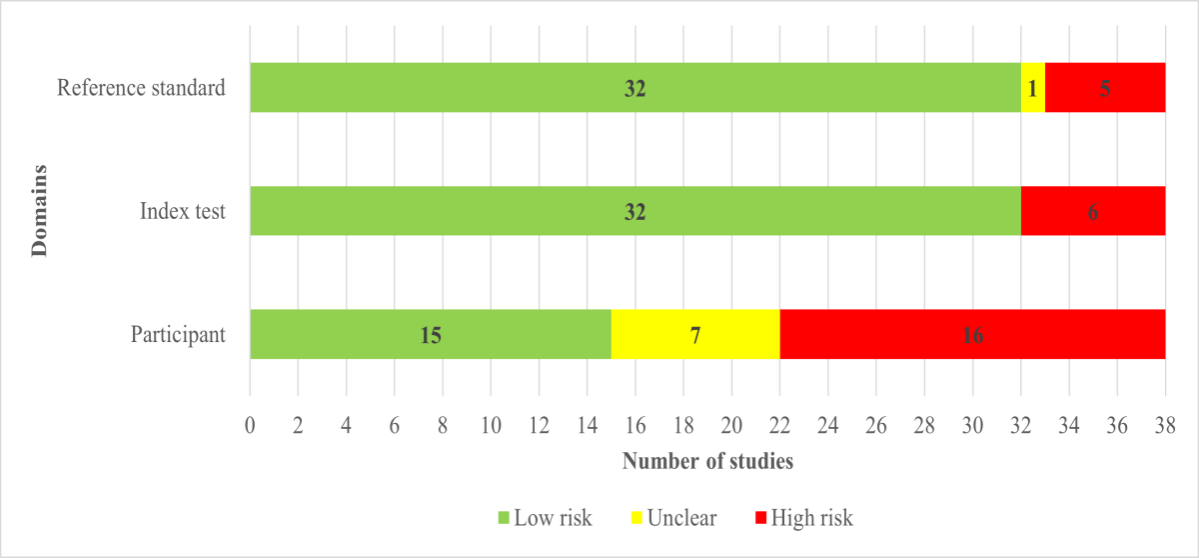
Results of the assessment of applicability concerns in the included studies.

Nearly half of the included studies (17/38, 45%) reported comprehensive details to determine whether an appropriate consecutive or random sample of eligible participants was used. Over half of the studies (22/38, 58%) avoided inappropriate exclusions. A substantial majority, 30 (79%) out of 38 studies, ensured a balanced number of patients across subgroups. In addition, around two-thirds (25/38, 66%) of the studies reported a sufficient sample size. Consequently, a little less than half of the studies (16/38, 42%) were assessed as having a low risk of bias in the “selection of participants” domain, as shown in Figure 2. In terms of matching participants to the predefined requirements in the review question, a low level of concern was identified in nearly 40% (15/38, 40%) of the included studies, as shown in Figure 3.

A substantial majority of the included studies comprehensively detailed their AI models, with 34 (89%) out of 38 studies providing thorough descriptions. Almost all, 35 (92%) out of 38 studies, clearly reported the features (predictors) used. Moreover, an overwhelming majority, 36 (95%) out of 38 studies, ensured that these features were sourced without prior knowledge of the outcome data. Consistency in feature assessment across participants was observed in 35 (92%) out of 38 studies. Consequently, the potential for bias in the “index test” domain was assessed as low in the vast majority of the studies (32/38, 84%), as shown in Figure 2. In addition, 32 (84%) out of 38 studies were found to have minimal concerns regarding the alignment between the model’s predictors and the review question’s criteria, as illustrated in Figure 3.

In most of the included studies (32/38, 84%), the outcome of interest, specifically sleep apnea, was consistently assessed using appropriate methodologies. Nearly all studies (37/38, 97%) defined and determined the outcome in a uniform manner for all participants. An overwhelming majority of the studies (36/38, 95%) determined the outcome without prior knowledge of the predictor information. In a substantial portion of the studies (33/38, 87%), the diagnostic test was conducted for an appropriate duration to ensure accurate results. As a result, the potential for bias in the “reference standard” domain was deemed low in the vast majority of the studies (32/38, 84%), as shown in Figure 2. In addition, the same number of studies (32/38, 84%) showed minimal concerns regarding any discrepancies between the outcome’s definition, timing, or determination and the review question’s criteria, as indicated in Figure 3.

Finally, a significant majority of the studies (34/38, 89%) ensured the inclusion of all enrolled participants in the data analysis. A substantial number of these studies (32/38, 84%) executed proper data preprocessing. Similarly, a high proportion (34/38, 89%) adopted suitable measures to evaluate the performance of their models. Nearly half of the studies (17/38, 45%) demonstrated an appropriate split among training, validation, and test sets. However, the risk of bias in the validation methods used by the remaining studies remained unclear due to insufficient information being provided. Consequently, slightly more than half of the studies (20/38, 53%) were deemed to have a low risk of bias in the “analysis” domain, as indicated in Figure 2. A detailed breakdown of the “risk of bias” and “applicability concerns” for each domain in every study is available in Multimedia Appendix 8.

### Results of the Studies

As mentioned earlier, meta-analyses were carried out to pool results related to 4 outcomes: detection of apnea events in respiration, identification of types of apnea events in respiration, detection of patients with sleep apnea, and estimation of the severity of sleep apnea. The following subsections present the results of the meta-analyses for each outcome.

#### Apnea Events in Respiration

##### Accuracy

We conducted meta-analyses of 36 estimates of accuracy derived from 2,702,305 respiratory events across 17 (45%) of the 38 studies (Table 4). The pooled mean accuracy of these estimates was 0.893 (95% CI 0.82-0.94). The meta-analyzed evidence exhibited considerable statistical heterogeneity (*P*<.001; *I*^2^=100%). Further, Table 4 shows that there is a statistically significant difference in the pooled mean accuracy between subgroups in the “algorithms” group (*P*<.001) and “type of algorithms” group (*P*=.02), whereas no statistically significant difference (*P*>.05) was found in the pooled mean accuracy between subgroups in the remaining groups.

**Table 4:** Pooled mean estimates of accuracy in detecting respiratory events by several factors.

Groups					Studies (N=38), n^a^ (%)		Sample size, N		Accuracy (%), range		Pooled mean accuracy (%; 95% CI)		Heterogeneity measures							Test for subgroup differences, *P* value	
													Tau^2^		Q (*P* value)		*I*^2^ (%)				
**Algorithm**																					*<.001* ^b^
	Convolutional neural network			9 (24)		437,593		0.76-0.97		0.884 (0.84-0.92)		0.0102		8523.6 (<.001)		100					
	Recurrent neural networks			6 (16)		665,091		0.73-0.92		0.848 (0.79-0.90)		0.0086		18,227 (<.001)		100					
	Random forest			4 (11)		807,225		0.81-0.96		0.867 (0.77-0.94)		0.0154		41,658 (<.001)		100					
	Support vector machine			3 (8)		245,745		0.79-0.84		0.807 (0.77-0.84)		0.0014		221.4 (<.01)		99					
	K-nearest neighbors			3 (8)		233,647		0.69-0.77		0.736 (0.69-0.78)		0.0019		78.1 (<.01)		97					
	AdaBoost			2 (5)		5629		0.71-0.72		0.716 (0.71-0.73)		0.0000		0.99 (.32)		0					
	Multilayer perceptron			2 (5)		242,505		0.80-0.81		0.804 (0.79-0.86)		0.0001		11.3 (<.01)		91					
	Quadratic discriminant analysis			2 (5)		17,727		0.60-0.73		0.664 (0.53-0.79)		0.0103		218.8 (<.01)		100					
**Type of algorithm**																					*.02*
	Machine learning			18 (47)		1,334,180		0.60-0.97		0.831 (0.65-0.92)		0.1926		289,302.4 (<.001)		100					
	Deep learning			18 (47)		1,368,125		0.73-1.00		0.899 (0.82-0.94)		0.2659		160,871.2 (<.001)		100					
**Sample size, n**																					.93
	<100			24 (63)		326,322		0.60-1.00		0.885 (0.74-0.95)		0.4783		111,302.0 (<.001)		100					
	100-200			3 (8)		276,572		0.82-0.93		0.896 (0.82-0.95)		0.0086		2658.6 (<.001)		100					
	<200			9 (24)		2,099,411		0.77-0.97		0.907 (0.75-0.97)		0.1238		314,844.0 (<.001)		100					
**Type of sleep apnea**																					>.99
	All			34 (89)		2,635,188		0.60-1.00		0.893 (0.80-0.94)		0.3616		457,420.0 (<.001)		96					
	Obstructive sleep apnea			2 (5)		67,117		0.88-0.90		0.892 (0.87-0.91)		0.0007		25.4 (<.001)		100					
**Status of the WD^c^**																					.05
	Commercial			22 (58)		2,581,505		0.69-0.97		0.844 (0.78-0.89)		0.0705		370,236.6 (<.001)		100					
	Noncommercial			14 (37)		120,800		0.60-1.00		0.947 (0.80-0.99)		0.6243		73,404.3 (<.001)		100					
**Placement of the WD**																					.61
	Chest			14 (37)		719,055		0.60-0.97		0.845 (0.64-0.94)		0.2173		143,429.2 (<.001)		100					
	Abdomen			7 (18)		127,375		0.73-1.00		0.951 (0.13-0.99)		1.4650		84,686.0 (<.001)		100					
	Chest and abdomen			3 (8)		261,949		0.76-0.93		0.880 (0.76-0.96)		0.0186		2849.4 (<.001)		100					
	Wrist			3 (8)		113,432		0.82-0.88		0.841 (0.80-0.88)		0.0024		790.1 (<.001)		100					
**Data set size, n**																					.90
	<10,000			12 (32)		34,088		0.60-0.97		0.863 (0.39-0.98)		0.4747		1136.8 (<.001)		100					
	10,000-50,000			11 (29)		173,171		0.73-1.00		0.907 (0.69-0.97)		0.6016		89,963.2 (<.001)		100					
	>50,000			10 (26)		2,218,474		0.73-0.97		0.881 (0.71-0.95)		0.1391		343,840.9 (<.001)		100					
**Data type**																					.30
	Respiration data			5 (13)		217,684		0.69-1.00		0.962 (0.53-1.0)		0.9962		56,612.8 (<.001)		100					
	HR^d^ data			2 (5)		27,735		0.82-0.90		0.864 (0.77-0.94)		0.0078		240.24 (<.001)		100					
	Respiration data and HR data			6 (16)		104,439		0.73-0.84		0.813 (0.77-0.85)		0.0116		852.8 (<.001)		100					
	Respiration data and SpO_2_^e^			10 (26)		2,108,997		0.76-0.97		0.896 (0.75-0.96)		0.1341		319,067.7 (<.001)		100					
	Respiration data, HR data, and body movement			12 (32)		238,494		0.60-0.88		0.787 (0.69-0.86)		0.0196		9958.4 (<.001)		100					
**Ground truth**																					.47
			Polysomnography	17 (45)		971,896		0.69-0.97		0.877 (0.81-0.92)		0.1433		185,452.6 (<.001)		100					
			WD	9 (24)		1,511,429		0.76-1.00		0.949 (0.19-1.0)		1.4613		148,018.6 (<.001)		100					
			Experiment context	10 (26)		218,980		0.60-0.93		0.856 (0.45-0.97)		0.2302		13,488.7 (<.001)		100					
**Validation method**																					.31
		K-fold cross-validation		12 (32)		2,173,814		0.69-0.97		0.835 (0.64-0.93)		0.1643		347,304 (<.001)		100					
		Train-test split		24 (63)		528,491		0.60-1.00		0.911 (0.82-0.96)		0.3667		106,748.9 (<.001)		100					
Overall accuracy					36 (95)		2,702,305		0.60-1.0		0.893 (0.82-0.94)		0.3130		457,567.0 (<.001)		100		—^f^		

^a^Many studies were included >1 time in most meta-analyses, given that the studies assessed the performance of >1 algorithm.

^b^Italicized values are statistically significant (*P*<.05).

^c^WD: wearable device.

^d^HR: heart rate.

^e^SpO_2_: blood oxygen saturation.

^f^Not applicable.

##### Sensitivity

As shown in Table 5, meta-analyses were carried out on 22 estimates of sensitivity derived from 872,443 respiratory events across 15 (39%) of the 38 studies. The pooled mean sensitivity of these estimates was 0.793 (95% CI 0.67-0.87). The meta-analyzed evidence has considerable statistical heterogeneity (*P*<.001; *I*^2^=100%). With regard to subgroup analyses, there was no statistically significant difference in the pooled mean sensitivity between subgroups in all groups.

**Table 5:** Pooled mean estimates of sensitivity in detecting respiratory events by several factors.

Groups			Studies, n^a^ (%)		Sample size, N		Sensitivity (%), range		Pooled mean sensitivity (%; 95% CI)		Heterogeneity measures							Test for subgroup difference, *P* value	
											Tau^2^		Q (*P* value)		*I*^2^ (%)				
**Algorithm**																			.39
	Convolutional neural network	8 (21)		107,274		0.25-0.94		0.752 (0.56-0.90)		0.0862		14,383.9 (<.001)		100					
	Recurrent neural networks	6 (16)		279,369		0.68-0.89		0.799 (0.72-0.86)		0.0119		7460.8 (<.001)		100					
	Random forest	2 (5)		141,601		0.68-0.80		0.737 (0.61-0.84)		0.0092		1628.9 (<.001)		100					
	K-nearest neighbors	2 (5)		114,368		0.30-0.69		0.499 (0.15-0.85)		0.0794		228.8 (<.01)		100					
**Type of algorithm**																			.80
	Machine learning	6 (16)		370,337		0.30-0.80		0.682 (0.50-0.80)		0.0696		3872.9 (<.001)		100					
	Deep learning	16 (42)		502,106		0.25-0.98		0.819 (0.69-0.90)		0.1397		42,230.7 (<.001)		100					
**Sample size, n**																			.41
	<100	10 (26)		41,761		0.25-0.98		0.813 (0.58-0.92)		0.1676		4692.1 (<.001)		100					
	100-200	3 (8)		57,022		0.70-0.87		0.801 (0.70-0.89)		0.0113		658.0 (<.001)		100					
	<200	9 (24)		773,660		0.44-0.94		0.718 (0.38-0.89)		0.0802		55,208.4 (<.001)		100					
**Type of sleep apnea**																			.99
	All	20 (53)		862,060		0.25-0.98		0.791 (0.67-0.87)		0.1006		58,431.0 (<.001)		100					
	Obstructive sleep apnea	2 (5)		10,383		0.44-0.93		0.724 (0.18-1.0)		0.1709		2217.2 (<.001)		100					
**Status of the WD^b^**																			.05
	Commercial	16 (42)		848,034		0.30-0.94		0.726 (0.61-0.81)		0.0214		58,113.7 (<.001)		100					
	Noncommercial	6 (16)		24,409		0.25-0.98		0.830 (0.60-0.97)		0.1051		1790.4 (<.001)		100					
**Placement of the WD**																			.36
	Chest	6 (16)		58,175		0.30-0.93		0.745 (0.45-0.89)		0.0079		1621.1 (<.001)		100					
	Chest and abdomen	3 (8)		55,783		0.78-0.87		0.826 (0.77-0.87)		0.0032		285.5 (<.001)		99					
	Wrist	3 (8)		18,548		0.44-0.70		0.617 (0.44-0.78)		0.0238		1316.3 (<.001)		100					
**Data set size, n**																			.63
	<10,000	4 (11)		1657		0.30-0.94		0.796 (0.71-0.97)		0.1338		191.7 (<.001)		99					
	10,000-50,000	5 (13)		13,137		0.25-0.98		0.768 (0.48-0.96)		0.1224		2218.8 (<.001)		100					
	>50,000	10 (26)		800,627		0.44-0.94		0.718 (0.48-0.86)		0.0523		57,265.8 (<.001)		100					
**Data type**																			.41
	Respiration data	5 (13)		35,749		0.30-0.98		0.888 (0.37-0.98)		0.3566		2046.3 (<.001)		100					
	HR^c^ data	2 (5)		7956		0.70-0.93		0.835 (0.56-0.99)		0.0499		600.2 (<.001)		100					
	Respiration data and SpO_2_^d^	10 (26)		787,542		0.68-0.94		0.814 (0.73-0.87)		0.0876		52,794.7 (<.001)		100					
	Respiration data, HR data, and body movement	4 (11)		40,457		0.44-0.80		0.658 (0.50-0.80)		0.0253		1688.2 (<.001)		100					
**Ground truth**																			.29
	Polysomnography	11 (29)		95,891		0.25-0.94		0.726 (0.53-0.85)		0.2373		7131.5 (<.001)		100					
	WD	9 (24)		742,136		0.69-0.98		0.900 (0.55-0.98)		0.1081		51,466.2 (<.001)		100					
	Experiment context	2 (5)		34,416		0.80-0.82		0.813 (0.79-0.83)		0.0002		2.47 (.12)		60					
**Validation method**																			.36
	K-fold cross-validation	12 (32)		795,959		0.30-0.94		0.743 (0.59-0.85)		0.0253		54,250.9 (<.001)		100					
	Train-test split	10 (26)		76,484		0.25-0.98		0.770 (0.61-0.90)		0.0765		8020.8 (<.001)		100					
Overall sensitivity			22 (58)		872,443		0.25-0.98		0.793 (0.67-0.87)		0.1196		62,433.8 (<.001)		100		—^e^		

^a^Many studies were included >1 time in most meta-analyses, given that the studies assessed the performance of >1 algorithm.

^b^WD: wearable device.

^c^HR: heart rate.

^d^SpO_2_: blood oxygen saturation.

^e^Not applicable.

##### Specificity

Meta-analyses were performed to pool 22 estimates of specificity derived from 1,699,503 respiratory events across 15 (39%) of the 38 studies (Table 6). The pooled mean specificity of these estimates was 0.946 (95% CI 0.88-0.98). There was considerable statistical heterogeneity (*P*<.001; *I*^2^=100%) in the meta-analyzed studies. We also found a statistically significant difference in the pooled mean specificity between subgroups in the “status of wearable device” group (*P*=.01), while there was no statistically significant difference (*P*>.05) in the pooled mean specificity between subgroups in the rest of the groups.

**Table 6:** Pooled mean estimates of specificity in detecting respiratory events by several factors.

Groups		Studies, n^a^ (%)	Sample size, N	Specificity (%), range	Pooled mean specificity (%; 95% CI)	Heterogeneity measures				Test for subgroup differences, *P* value	
						Tau^2^	Q (*P* value)	*I*² (%)			
**Algorithm**											.14
	Convolutional neural network	8 (21)	298,315	0.72-0.99	0.932 (0.87-0.98)	0.0236	6463.7 (<.001)	100			
	Recurrent neural networks	6 (16)	385,722	0.76-0.95	0.870 (0.81-0.92)	0.0090	11,722.7 (<.001)	100			
	Random forest	2 (5)	647,897	0.85-0.98	0.930 (0.74-1.00)	0.0374	28,107.9 (<.001)	100			
	K-nearest neighbors	2 (5)	116,039	0.76-0.86	0.812 (0.71-0.90)	0.0079	127.1 (<.01)	99			
**Type of algorithm**											.10
	Machine learning	6 (16)	879,975	0.70-0.98	0.910 (0.38-0.99)	0.5700	259,327.0 (<.001)	100			
	Deep learning	16 (42)	819,528	0.72-1.00	0.949 (0.87-0.98)	0.6454	158,624.8 (<.001)	100			
**Sample size, n**											.94
	<100	10 (26)	154,202	0.70-1.00	0.951 (0.79-0.99)	1.0272	123,494.7 (<.001)	100			
	100-200	3 (8)	219,550	0.84-0.95	0.922 (0.85-0.97)	0.0112	2860.8 (<.001)	100			
	<200	9 (24)	1,325,751	0.85-0.98	0.949 (0.81-0.99)	0.2549	340,888.9 (<.001)	100			
**Type of sleep apnea**											.97
	All	20 (53)	1,642,769	0.70-1.00	0.947 (0.86-0.98)	0.7459	487,691.1 (<.001)	100			
	Obstructive sleep apnea	2 (5)	56,734	0.94-0.95	0.943 (0.94-0.95)	0.0100	3.0 (.09)	66			
**Status of the WD^b^**											*.01* ^c^
	Commercial	16 (42)	1,629,032	0.70-0.98	0.887 (0.79-0.94)	0.2257	413,387.5 (<.001)	100			
	Noncommercial	6 (16)	70,471	0.85-1.00	0.969 (0.92-1.00)	0.0181	2607.4 (<.001)	100			
**Placement of the WD**											.97
	Chest	6 (16)	634,960	0.70-0.98	0.900 (0.65-0.97)	0.3678	157,806.3 (<.001)	100			
	Chest and abdomen	3 (8)	206,166	0.72-0.95	0.893 (0.73-0.99)	0.0370	4312.8 (<.001)	100			
	Wrist	3 (8)	94,884	0.84-0.94	0.885 (0.82-0.94)	0.0074	2106.2 (<.001)	100			
**Data set size, n**											.64
	<10,000	4 (11)	6511	0.70-0.99	0.923 (0.06-1.00)	0.8131	416.7 (<.001)	100			
	10,000-50,000	5 (13)	55,595	0.72-1.00	0.937 (0.81-1.00)	0.0482	9402.4 (<.001)	100			
	>50,000	10 (26)	1,417,847	0.76-0.98	0.888 (0.84-0.93)	0.1031	93,530.5 (<.001)	100			
**Data type**											.37
	Respiration data	5 (13)	181,935	0.70-1.00	0.977 (0.61-1.00)	1.1621	53,359.8 (<.001)	100			
	HR^d^ data	2 (5)	19,779	0.86-0.95	0.908 (0.80-0.98)	0.0121	217.8 (<.001)	99			
	Respiration data and SpO_2_^e^	10 (26)	1,321,455	0.72-0.98	0.925 (0.72-0.98)	0.3791	348,529.0 (<.001)	100			
	Respiration data, HR data, and body movement	4 (11)	172,117	0.76-0.94	0.864 (0.67-0.95)	0.0117	9381.8 (<.001)	100			
**Ground truth**											.90
	Polysomnography	11 (29)	771,566	0.70-1.00	0.948 (0.86-0.98)	0.5619	202,709.3 (<.001)	100			
	WD	9 (24)	769,293	0.72-1.00	0.957 (0.11-1.00)	1.8198	103,183.0 (<.001)	100			
	Experiment context	2 (5)	158,644	0.85-0.95	0.908 (0.78-0.98)	0.0158	293.1 (<.001)	100			
**Validation method**											.10
	K-fold cross-validation	12 (32)	1,377,855	0.70-0.98	0.866 (0.61-0.96)	0.3674	401,832.4 (<.001)	100			
	Train-test split	10 (26)	321,648	0.84-1.00	0.947 (0.90-0.98)	0.0178	8482.6 (<.001)	100			
Overall specificity		22 (58)	1,699,503	0.70-1.00	0.946 (0.88-0.98)	0.6373	487,706.6 (<.001)	100	—^f^		

^a^Many studies were included more than one time in all meta-analyses given that the studies assessed the performance of more than one algorithm.

^b^WD: wearable device.

^c^Italicized values are statistically significant (*P*<.05).

^d^HR: heart rate.

^e^SpO_2_: blood oxygen saturation.

^f^Not applicable.

#### Type of Apnea Events in Respiration

We conducted meta-analyses of 6 estimates of accuracy derived from 637,250 respiratory events across 6 (16%) of the 38 studies (Table 7). The pooled mean accuracy of these estimates was 0.815 (95% CI 0.64-0.94). The meta-analyzed studies exhibited considerable statistical heterogeneity (*P*<.001; *I*^2^=100%). In addition, there was a statistically significant difference in the pooled mean accuracy between subgroups in the “data type” group (*P*=.001), while no statistically significant difference (*P*>.05) was found in the pooled mean accuracy between subgroups in the remaining groups.

**Table 7:** Pooled mean estimates of accuracy in detecting the type of respiratory events by several factors.

Groups			Studies, n (%)		Sample size, N		Accuracy (%), range		Pooled mean accuracy (%; 95% CI)		Heterogeneity measures							Test for subgroup differences, *P* value	
											Tau^2^		Q (*P* value)		*I*² (%)				
**Algorithm**																			.76
	Convolutional neural network	4 (11)		459,163		0.40-0.97		0.829 (0.55-0.99)		0.0973		26,906.1 (<.001)		100					
	Long short-term memory	2 (5)		178,087		0.73-0.84		0.788 (0.68-0.88)		0.0073		2299.2 (<.001)		100					
**Sample size, n**																			.27
	≤100	3 (8)		309,033		0.74-0.97		0.892 (0.73-0.98)		0.0328		21,243.3 (<.001)		100					
	>100	3 (8)		328,217		0.40-0.88		0.724 (0.41-0.95)		0.0824		19,923.0 (<.001)		100					
**Status of the WD^a^**																			.25
	Commercial	4 (11)		578,076		0.40-0.93		0.759 (0.51-0.94)		0.0735		41,330.6 (<.001)		100					
	Noncommercial	2 (5)		59,174		0.84-0.97		0.909 (0.74-0.99)		0.0249		30.9 (<.01)		97					
**Data type**																			*.001* ^b^
	Respiration data	2 (5)		189,970		0.93-0.97		0.944 (0.91-0.97)		00022		3.7 (.05)		73					
	Respiration data and SpO_2_^c^	2 (5)		308,810		0.84-0.88		0.857 (0.81-0.90)		0.0018		697.4 (<.01)		100					
	Respiration data, HR^d^ data, and body movement	2 (5)		138,470		0.40-0.74		0.574 (0.25-0.87)		0.0590		7875.2 (<.001)		100					
**Ground truth**																			.19
	Polysomnography	4 (11)		197,644		0.40-0.97		0.762 (0.49-0.95)		0.0822		12,665.3 (<.001)		100					
	Nonpolysomnography	2 (5)		439,606		0.88-0.93		0.905 (0.85-0.95)		0.0039		3353.7 (<.001)		100					
**Validation method**																			.97
	K-fold cross-validation	2 (5)		368,849		0.74-0.88		0.812 (0.66-0.93)		0.0164		10,579.9 (<.001)		100					
	Train-test split	4 (11)		268,401		0.40-0.97		0.818 (0.54-0.98)		0.0954		28,010.1 (<.001)		100					
Overall accuracy			6 (16)		637,250		0.40-0.97		0.815 (0.64-0.94)		0.0603		41,608.1 (<.001)		100		—^e^		

^a^WD: wearable device.

^b^Italicized values are statistically significant (*P*<.05).

^c^SpO_2_: blood oxygen saturation.

^d^HR: heart rate.

^e^Not applicable.

#### Patients With Sleep Apnea

##### Accuracy

We carried out meta-analyses of 13 estimates of accuracy derived from 2015 participants across 13 (34%) of the 38 studies (Table 8). The pooled mean accuracy of these estimates was 0.869 (95% CI 0.81-0.92). The meta-analyzed estimates showed considerable statistical heterogeneity (*P*<.001; *I*^2^=100%). Further, there was a statistically significant difference in the pooled mean accuracy between subgroups in the “type of sleep apnea” group (*P*=.049). However, no statistically significant difference (*P*>.05) was found in the pooled mean accuracy between subgroups in the remaining groups.

**Table 8:** Pooled mean estimates of accuracy in detecting sleep apnea by several factors.

Groups			Studies, n (%)		Sample size, N		Accuracy (%), range		Pooled mean accuracy (%; 95% CI)		Heterogeneity measures							Test for subgroup differences, *P* value	
											Tau^2^		Q (*P* value)		*I*² (%)				
**Type of algorithm**																			.20
	Machine learning	3 (8)		1141		0.88-0.92		0.896 (0.87-0.92)		0.0003		2.2 (.34)		7					
	Deep learning	9 (24)		678		0.71-1.00		0.849 (0.76-0.92)		0.0226		55.4 (<.01)		86					
**Sample size, n**																			.20
	≤100	8 (21)		496		0.71-0.96		0.838 (0.77-0.90)		0.0095		23.3 (<.01)		70					
	>100	5 (13)		1519		0.75-1.00		0.905 (0.81-0.97)		0.0224		45.5 (<.01)		91					
**Type of sleep apnea**																			*.049* ^a^
	All	6 (16)		671		0.78-1.00		0.920 (0.84-0.97)		0.0163		24.4 (<.01)		80					
	Obstructive sleep apnea	7 (18)		1344		0.71-0.91		0.823 (0.76-0.88)		0.0091		40.5 (<.01)		85					
**Status of the WD^b^**																			.18
	Commercial	9 (24)		1632		0.71-0.96		0.841 (0.78-0.89)		0.0098		49.5 (<.01)		84					
	Noncommercial	4 (11)		383		0.78-1.00		0.923 (0.81-0.99)		0.0252		22.4 (<.01)		87					
**Placement of the WD**																			.17
	Wrist	4 (11)		982		0.74-0.96		0.840 (0.73-0.93)		0.0165		28.4 (<.01)		98					
	Finger	3 (8)		212		0.71-0.86		0.805 (0.70-0.89)		0.0070		5.99 (.05)		67					
	Chest and abdomen	3 (8)		147		0.86-1.00		0.949 (0.83-1.00)		0.0230		10.6 (<.01)		81					
**Data type**																			.09
	Respiration data and SpO_2_^c^	4 (11)		556		0.86-1.00		0.938 (0.86-0.99)		0.0139		13.4 (<.01)		78					
	Respiration data, HR^d^ data, and body movement	4 (11)		408		0.71-0.91		0.840 (0.74-0.92)		0.0111		17.1 (<.01)		82					
**Ground truth**																			.12
	Polysomnography	11 (29)		1908		0.71-1.00		0.878 (0.82-0.93)		0.0164		71.1 (<.01)		86					
	WD	2 (5)		107		0.74-0.86		0.789 (0.67-0.89)		0.0036		1.6 (.20)		38					
**Validation method**																			.91
	K-fold cross-validation	4 (11)		1177		0.78-0.91		0.873 (0.82-0.92)		0.0035		8.2 (.04)		63					
	Train-test split	7 (18)		698		0.71-1.00		0.880 (0.78-0.95)		0.0267		59.2 (<.01)		90					
	Leave-one-out cross-validation	2 (5)		140		0.74-0.92		0.839 (0.64-0.97)		0.0240		7.7 (<.01)		87					
Overall accuracy			13 (34)		2015		0.71-1.00		0.869 (0.81-0.92)		0.0156		80.3 (<.001)		100		—^e^		

^a^Italicized values are statistically significant (*P*<.05).

^b^WD: wearable device.

^c^SpO_2_: blood oxygen saturation.

^d^HR: heart rate.

^e^Not applicable.

##### Sensitivity

As shown in Table 9, meta-analyses were carried out on 13 estimates of sensitivity derived from 1580 participants across 13 (34%) of the 38 studies. The pooled mean sensitivity of these estimates was 0.938 (95% CI 0.89-0.97). The meta-analyzed evidence has considerable statistical heterogeneity (*P*<.001; *I*^2^=82%). With regards to subgroup analyses, there was no statistically significant difference in the pooled mean sensitivity between subgroups in all groups except for the “placement of wearable device” group (*P*<.001).

**Table 9:** Pooled mean estimates of sensitivity in detecting sleep apnea by several factors.

Groups			Studies, n (%)		Sample size, N		Sensitivity (%), range		Pooled mean sensitivity (%; 95% CI)		Heterogeneity measures								Test for subgroup differences, *P* value	
											Tau^2^		Q (*P* value)		*I*² (%)					
**Type of algorithm**																				.78
	Machine learning	3 (8)		921		0.89-0.98		0.926 (0.88-0.98)		0.0038		6.9 (.03)		71						
	Deep learning	9 (24)		485		0.77-1.00		0.942 (0.87-0.99)		0.0256		59.0 (<.01)		86						
**Sample size, n**																				.50
	≤100	8 (21)		363		0.77-1.00		0.953 (0.90-0.99)		0.0154		28.0 (<.01)		75						
	>100	5 (13)		1217		0.77-1.00		0.917 (0.83-0.97)		0.0196		33.9 (<.01)		88						
**Type of sleep apnea**																				.06
	All	6 (16)		456		0.90-1.00		0.959 (0.93-1.00)		0.0059		13.0 (.02)		62						
	Obstructive sleep apnea	7 (18)		1124		0.77-1.00		0.903 (0.83-0.96)		0.0179		37.8 (<.01)		84						
**Status of the WD^a^**																				.06
	Commercial	9 (24)		1254		0.77-1.00		0.916 (0.85-0.96)		0.0170		43.2 (<.01)		81						
	Noncommercial	4 (11)		326		0.93-1.00		0.974 (0.93-1.00)		0.0048		7.5 (.06)		60						
**Placement of the WD**																				*<.001* ^b^
	Wrist	4 (11)		833		0.77-0.90		0.837 (0.76-0.91)		0.0073		16.1 (<.01)		81						
	Finger	3 (8)		148		0.92-1.00		0.966 (0.90-1.00)		0.0000		5.3 (.07)		62						
	Chest and abdomen	3 (8)		130		0.98-1.00		0.997 (0.97-1.00)		0.0083		1.1 (.59)		0						
**Data type**																				.39
	Respiration data and SpO_2_^c^	4 (11)		378		0.92-1.00		0.980 (0.93-1.00)		0.0080		11.0 (.01)		73						
	Respiration data, HR^d^ data, and body movement	4 (11)		322		0.92-0.95		0.954 (0.91-0.99)		0.0040		6.2 (.10)		52						
**Ground truth**																				.80
	Polysomnography	11 (29)		1495		0.77-1.00		0.941 (0.89-0.97)		0.0126		52.6 (<.01)		81						
	WD	2 (5)		85		0.77-1.00		0.917 (0.57-1.00)		0.0773		12.1 (<.01)		92						
**Validation method**																				.89
	K-fold cross-validation	4 (11)		941		0.89-1.00		0.944 (0.89-0.98)		0.0063		10.7 (.01)		72						
	Train-test split	7 (18)		527		0.77-1.00		0.941 (0.87-0.99)		0.0199		41.9 (<.01)		86						
	Leave-one-out cross-validation	2 (5)		112		0.77-0.98		0.896 (0.61-1.00)		0.0542		13.2 (<.01)		92						
Overall sensitivity			13 (34)		1580		0.77-1.00		0.938 (0.89-0.97)		0.0162		67.0 (<.001)		82			—^e^		

^a^WD: wearable device.

^b^Italicized values are statistically significant (*P*<.05).

^c^SpO_2_: blood oxygen saturation.

^d^HR: heart rate.

^e^Not applicable.

##### Specificity

Meta-analyses were performed to pool 13 estimates of specificity derived from 436 participants across 13 (34%) of the 38 studies (Table 10). The pooled mean specificity of these estimates was 0.752 (95% CI 0.63-0.86). There was considerable statistical heterogeneity (*P*<.001; *I*^2^=78%) in the meta-analyzed studies. Our subgroup meta-analyses showed that there was no statistically significant difference in the pooled mean specificity between subgroups in all groups.

**Table 10:** Pooled mean estimates of specificity in detecting sleep apnea by several factors.

Groups		Studies, n (%)	Sample size, N	Specificity (%), range	Pooled mean specificity (%; 95% CI)	Heterogeneity measures				Test for subgroup differences, *P* value	
						Tau^2^	Q (*P* value)	*I*² (%)			
**Type of algorithm**											.53
	Machine learning	3 (8)	220	0.60-0.88	0.796 (0.63-0.92)	0.0168	7.9 (.02)	75			
	Deep learning	9 (24)	194	0.29-1.00	0.735 (0.55-0.89)	0.0575	37.1 (<.01)	78			
**Sample size, n**											.21
	≤100	8 (21)	133	0.29-1.00	0.690 (0.48-0.87)	0.0615	35.8 (<.01)	80			
	>100	5 (13)	303	0.72-1.00	0.818 (0.72-0.90)	0.0077	11.6 (.02)	65			
**Type of sleep apnea**											.62
	All	6 (16)	220	0.29-0.89	0.810 (0.55-0.99)	0.0780	34.6 (<.01)	86			
	Obstructive sleep apnea	7 (18)	216	0.36-1.00	0.730 (0.64-0.81)	0.0068	12.3 (.06)	51			
**Status of the WD^a^**											.43
	Commercial	9 (24)	378	0.29-1.00	0.784 (0.66-0.89)	0.0295	32.9 (<.01)	76			
	Noncommercial	4 (11)	58	0.36-1.00	0.672 (0.39-0.91)	0.0497	11.3 (.01)	73			
**Placement of the WD**											.71
	Wrist	4 (11)	149	0.72-1.00	0.803 (0.62-0.94)	0.0309	10.9 (.01)	73			
	Finger	3 (8)	64	0.29-0.88	0.658 (0.29-0.89)	0.0727	11.5 (<.01)	83			
	Chest and abdomen	3 (8)	18	0.60-1.00	0.777 (0.47-0.99)	0.0157	2.7 (.26)	26			
**Data type**											.35
	Respiration data and SpO_2_^b^	4 (11)	179	0.60-1.00	0.855 (0.66-0.99)	0.0186	5.6 (.13)	47			
	Respiration data, HR^c^ data, and body movement	4 (11)	86	0.29-0.89	0.700 (0.46-0.90)	0.0411	11.5 (<.01)	74			
**Ground truth**											.53
	Polysomnography	11 (29)	414	0.29-1.00	0.756 (0.62-0.88)	0.0444	52.8 (<.01)	81			
	WD	2 (5)	22	0.67-0.75	0.691 (0.46-0.89)	0.0000	0.04 (.85)	0			
**Validation method**											.48
	K-fold cross-validation	4 (11)	236	0.36-0.88	0.713 (0.44-0.93)	0.0547	26.9 (<.01)	89			
	Train-test split	7 (18)	172	0.29-1.00	0.793 (0.72-0.86)	0.0456	22.9 (<.01)	74			
	Leave-one-out cross-validation	2 (5)	28	0.60-0.67	0.644 (0.45-0.82)	0.0000	0.13 (.72)	0			
Overall specificity		13 (34)	436	0.29-1.00	0.752 (0.63-0.86)	0.0366	54.5 (<.001)	78	—^d^		

^a^WD: wearable device.

^b^SpO_2_: blood oxygen saturation.

^c^HR: heart rate.

^d^Not applicable.

#### Severity of Sleep Apnea

##### Accuracy

We performed meta-analyses of 9 estimates of accuracy derived from 1661 participants across 9 (24%) of the 38 studies (Table 11). The pooled mean accuracy of these estimates was 0.651 (95% CI 0.54-0.75). The meta-analyzed studies exhibited considerable statistical heterogeneity (*P*<.001; *I*^2^=93%). In addition, there was a statistically significant difference in the pooled mean accuracy between subgroups in “type of sleep apnea” group (*P*=.03) and “data type” group (*P*=.01), while no statistically significant difference (*P*>.05) was found in the pooled mean accuracy between subgroups in the remaining groups.

**Table 11:** Pooled mean estimates of accuracy in detecting the severity of sleep apnea by several factors.

Groups		Studies, n (%)	Sample size, N	Accuracy (%), range	Pooled mean accuracy (%; 95% CI)	Heterogeneity measures				Test for subgroup difference, *P* value	
						Tau^2^	Q (*P* value)	*I*² (%)			
**Type of algorithm**											.28
	Machine learning	3 (8)	1141	0.63-0.80	0.716 (0.60-0.82)	0.0105	40.6 (<.01)	95			
	Deep learning	6 (16)	520	0.36-0.89	0.615 (0.46-0.76)	0.0318	47.8 (<.01)	90			
**Sample size, n**											.28
	≤100	4 (11)	274	0.36-0.71	0.584 (0.43-0.74)	0.0219	22.4 (<.01)	87			
	>100	5 (13)	1387	0.55-0.89	0.698 (0.56-0.82)	0.0252	72.9 (<.01)	95			
**Type of sleep apnea**											*.03* ^a^
	All	4 (11)	591	0.58-0.89	0.757 (0.62-0.87)	0.0196	20.3 (<.01)	85			
	Obstructive sleep apnea	5 (13)	1070	0.36-0.67	0.564 (0.46-0.66)	0.0111	23.3 (<.01)	83			
**Status of the WD^b^**											.08
	Commercial	7 (18)	1543	0.36-0.80	0.606 (0.50-0.71)	0.0219	87.9 (<.01)	93			
	Noncommercial	2 (5)	118	0.71-0.89	0.809 (0.60-0.95)	0.0181	6.2 (.01)	84			
**Placement of the WD**											.11
	Wrist	3 (8)	922	0.55-0.63	0.596 (0.54-0.65)	0.0011	3.5 (.18)	42			
	Finger	3 (8)	212	0.36-0.67	0.542 (0.35-0.72)	0.0235	16.5 (<.01)	88			
	Chest and abdomen	2 (5)	118	0.71-0.89	0.809 (0.60-0.95)	0.0219	6.2 (.01)	84			
**Data type**											*.01*
	Respiration data and SpO_2_^c^	3 (8)	527	0.71-0.89	0.807 (0.71-0.89)	0.0073	6.2 (.04)	68			
	Body movement, HR^d^ data, and SpO_2_	3 (8)	212	0.36-0.67	0.542 (0.35-0.72)	0.0235	16.5 (<.01)	88			
**Validation method**											.37
	K-fold cross-validation	2 (5)	1079	0.63-0.80	0.719 (0.53-0.87)	0.0195	40.6 (<.01)	98			
	Train-test split	6 (16)	520	0.36-0.89	0.615 (0.46-0.76)	0.0318	47.8 (<.01)	90			
Overall accuracy		9 (24)	1661	0.36-0.89	0.651 (0.54-0.75)	0.0243	106.1 (<.001)	93	—^e^		

^a^Italicized values are statistically significant (*P*<.05).

^b^WD: wearable device.

^c^SpO_2_: blood oxygen saturation.

^d^HR: heart rate.

^e^Not applicable.

#### Correlation Coefficient

As shown in Table 12, meta-analyses were carried out on 12 estimates of correlation coefficient (*r*) derived from 1266 participants across 12 (32%) of the 38 studies. The pooled mean *r* of these estimates was 0.877 (95% CI 0.82-0.92). The meta-analyzed evidence has considerable statistical heterogeneity (*P*<.001; *I*^2^=82%). With regard to subgroup analyses, there was a statistically significant difference in the pooled mean r between subgroups in the “placement of wearable device” group (*P*<.001) and the “data type” group (*P*<.001). However, no statistically significant difference (*P*>.05) was found in the pooled mean *r* between subgroups in the remaining groups.

**Table 12:** Pooled mean estimates of correlation coefficient (r) in detecting the severity of sleep apnea by several factors.

Groups			Studies, n (%)		Sample size, N		Correlation coefficient (%), range		Pooled mean correlation coefficient (%; 95% CI)		Heterogeneity measures							Test for subgroup differences, *P* value	
											Tau^2^		Q (*P* value)		*I*² (%)				
**Type of algorithm**																			.12
	Machine learning	3 (8)		526		0.90 to 0.96		0.922 (0.66 to0.98)		0.0987		25.6 (<.01)		92					
	Deep learning	9 (24)		740		0.64 to 0.91		0.856 (0.79 to0.90)		0.0552		53.1 (<.01)		85					
**Sample size, n**																			.79
	≤100	9 (24)		541		0.64 to 0.90		0.879 (0.85 to0.90)		0.0001		12.7 (.12)		37					
	>100	3 (8)		725		0.71 to 0.96		0.896 (0.12 to0.99)		0.2849		173.0 (<.01)		99					
**Type of sleep apnea**																			.54
	All	8 (21)		802		0.64 to 0.96		0.886 (0.81 to0.93)		0.0918		80.2 (<.01)		91					
	Obstructive sleep apnea	4 (11)		464		0.71 to 0.90		0.859 (0.68 to0.94)		0.759		34.7 (<.01)		91					
**Status of the WD^a^**																			.35
	Commercial	10 (26)		1177		0.64 to 0.96		0.881 (0.82 to 0.92)		0.0977		191.1 (<.01)		95					
	Noncommercial	2 (5)		89		0.84 to 0.86		0.856 (0.72 to 0.93)		0.0000		0.1 (.79)		0					
**Placement of the WD**																			*<.001* ^b^
	Wrist	2 (5)		316		0.71 to 0.91		0.833 (–0.99 to 1.00)		0.1948		20.1 (<.01)		95					
	Chest	2 (5)		452		0.87 to 0.96		0.929 (–0.98 to 1.00)		0.1765		16.7 (<.01)		94					
	Finger	3 (8)		212		0.89 to 0.90		0.894 (0.88 to0.91)		0.0000		0.1 (.93)		0					
	Neck	2 (5)		89		0.84 to 0.86		0.856 (0.72 to0.93)		0.0000		0.1 (.79)		0					
	Abdomen	2 (5)		158		0.89 to 0.90		0.894 (0.81 to 0.94)		0.0000		0.1 (.76)		0					
**Data type**																			<.001
	Respiration data	2 (5)		89		0.84 to 0.86		0.856 (0.72 to 0.93)		0.0000		0.1 (.79)		0					
	Respiration data and SpO_2_^c^	2 (5)		438		0.64 to 0.96		0.878 (–1.00 to 1.00)		0.6849		34.5 (<.01)		97					
	Respiration data and HR^d^ data	2 (5)		158		0.89 to 0.90		0.894 (0.81 to 0.94)		0.0000		0.1 (.76)		0					
	Respiration data, HR data, and body movement	3 (8)		369		0.71 to 0.91		0.844 (0.38 to 0.97)		0.1030		24.5 (<.01)		92					
	Body movement, HR data, and SpO_2_	3 (8)		212		0.89 to 0.90		0.894 (0.88 to 0.91)		0.0000		0.1 (.93)		0					
**Validation method**																			.90
	K-fold cross-validation	4 (11)		527		0.64 to 0.96		0.869 (0.49-0.97)		0.2315		59.3 (<.01)		95					
	Train-test split	7 (18)		450		0.71 to 0.91		0.877 (0.82-0.92)		0.0486		50.8 (<.01)		88					
Overall accuracy			12 (32)		1266		0.64 to 0.96		0.877 (0.82-0.92)		0.0828		194.5 (<.001)		94		—^e^		

^a^WD: wearable device.

^b^Italicized values are statistically significant (*P*<.05).

^c^SpO_2_: blood oxygen saturation.

^d^HR: heart rate.

^e^Not applicable.

## Discussion

### Principal Findings

This systematic review investigated how well wearable AI performs in detecting sleep apnea. Overall, the findings indicate that wearable AI demonstrated a performance that is deemed acceptable, although not optimal, for detecting sleep apnea. Specifically, wearable AI was able to correctly classify apnea events and nonapnea events in 89.3% of respiratory events. This performance was notably higher when using CNN in particular or deep learning algorithms in general. The superiority of CNN architectures can be attributed to their ability to capture the localized dependencies inherent in apnea patterns through convolution kernels. The meta-analyses conducted in this review revealed that wearable AI performed better in detecting nonapnea respiratory events (94.6%) compared to apnea respiratory events (79.3%). This could be linked to the training of AI models using an unrepresentative sample, wherein the number of nonapnea respiratory events (n=1,699,503) was approximately twice as high as the number of apnea respiratory events (n=872,443). This highlights the challenge of applying data balancing techniques for heterogeneous and time-dependent measurements, particularly evident in longitudinal recordings as observed in apnea studies.

Although the sensitivity of wearable AI in detecting apnea events in respiration remained unaffected by any moderating factors, the specificity was influenced by the status of the wearable device, where noncommercial devices exhibited higher specificity than commercial devices. This can be because all studies that used noncommercial wearable devices applied deep learning algorithms, whereas more than one-third (6/16, 38%) of studies that used commercial wearable devices applied ML algorithms (eg, random forest, AdaBoost, and k-nearest neighbors). Introducing scalable AI models, such as deep learning models, into commercial apnea detection applications presents challenges due to their computational expense and resource requirements, thereby complicating market penetration and impacting profit margins. However, recent advancements in tiny ML models and edge AI implementations offer potential solutions to mitigate these challenges. This review also demonstrated that wearable AI was able to correctly differentiate between different types of apnea events (eg, apnea, hypopnea, obstructive apnea, and central apnea) in 81.5% of respiratory events, and this performance was not influenced by any moderators. This can be attributed to the lack of studies (≤4) in all subgroup analyses related to this outcome.

In this review, wearable AI demonstrated 86.9% accuracy in correctly identifying patients with and patients without sleep apnea. This performance was notably higher when the wearable AI was used for detecting sleep apnea in general (92%) rather than OSA in particular (82.3%). This difference may be attributed to the fact that approximately 83% (5/6) of studies focusing on general sleep apnea detection used respiration data to develop the AI models. By contrast, only 29% (2/7) of studies concentrating on OSA detection incorporated respiration data. Given that respiration data are widely acknowledged as the most crucial indicator of sleep apnea, this disparity in use may explain the varying performance levels observed in the review.

Unlike apnea event detection, wearable AI exhibited superior performance in identifying patients with sleep apnea (93.8%) compared to those without sleep apnea (75.2%). This could be associated with the training of AI models using an unrepresentative sample, wherein the number of patients with sleep apnea (1580) was >3 times higher than the number of patients without sleep apnea (436). The specificity of wearable AI in detecting sleep apnea was not affected by any moderator, while its sensitivity was higher when wearable devices were placed on both the chest and abdomen in comparison with other placements (wrist or fingers). This moderation effect could be attributed to the fact that all studies that placed wearable devices on both the chest and abdomen focused on detecting sleep apnea in general, while 6 (86%) out of 7 studies that placed wearable devices in other places focused on detecting OSA in particular. Further, all studies that placed wearable devices on other body parts used commercial wearable devices, whereas only 1 of the studies that placed wearable devices on both the chest and abdomen used commercial wearable devices.

Our meta-analyses also revealed that wearable AI accurately differentiated between various levels of sleep apnea severity (normal, mild, moderate, and severe) in 65.1% of cases. This performance was higher when the wearable AI was used for detecting the severity of sleep apnea in general rather than OSA in particular. This could be linked to the fact that all studies that aimed to detect OSA used commercial devices that were placed on either fingers or wrists, while two-thirds of the studies that focused on sleep apnea in general used noncommercial devices that were placed on both the abdomen and chest. This performance was also higher when the model was developed using both respiration and oxygen saturation data in comparison with using a combination of body movement, HR, and oxygen saturation data. This could be associated with the fact that all studies using the combination of body movement, HR, and oxygen saturation data focused on the detection of OSA using commercial devices placed on fingers, while all studies using both respiration and oxygen saturation data focused on detecting any sleep apnea type using noncommercial devices (in 2, 67% out 3 studies) placed on the abdomen and chest.

Finally, the accuracy of wearable AI in estimating the severity of sleep apnea (ie, the apnea-hypopnea index score) reached 87.7%. This accuracy was higher when the wearable device was placed on the chest and when using both respiration and HR data or a combination of HR, oxygen saturation, and body movement.

### Research and Practical Implications

Our analysis revealed that wearable AI shows promise in identifying sleep apnea, distinguishing its type, and gauging its severity; however, it is not yet ready for widespread use in clinical practices for 3 reasons. First, its current performance falls below the optimal level. Second, only 9 (24%) of the 38 studies were judged to have a low risk of bias in all domains. Third, heterogeneity between studies was considerable in most meta-analyses. Therefore, we cannot propose the use of wearable AI as a replacement for traditional sleep assessments (eg, polysomnography and home sleep apnea testing), but we recommend that wearable AI be used in conjunction with these assessments, taking into account factors such as cost-effectiveness and practical challenges in real-world implementation.

Among all wearable devices used in the included studies, only 1 was specifically designed for diagnosing sleep and monitoring sleep health and obtained clearance from the US Food and Drug Administration. This may be due to a shortage of such wearable devices in the market or a scarcity of studies evaluating them. We urge manufacturers of wearable devices to extend their focus beyond evaluating sleep quality and incorporate AI into their devices for identifying sleep apnea, its various types, and its severity. Further, researchers should pay more attention to such wearable devices in their future studies. The main challenge of conducting such studies is the cost of such wearable devices.

Our meta-analyses indicate that the performance of wearable AI was notably higher when using CNN in particular or deep learning algorithms in general. Therefore, we recommend that manufacturers of wearable devices and researchers prioritize these techniques during the development of devices intended for the detection of sleep apnea. However, obtaining large, high-quality, and standardized data sets for training and validating CNN or deep learning models can be challenging.

Our meta-analysis suggests the need for implementing AI on the edge through specially crafted tiny ML modules with federated learning protocols. Such an approach not only enhances performance metrics but also addresses critical considerations regarding resource efficiency, latency reduction, and privacy preservation inherent in commercial apnea detection systems. However, implementing AI on wearable devices, especially with tiny ML modules, poses challenges related to hardware constraints, such as limited processing power, limited memory, and high energy consumption. Further, ensuring that AI algorithms can run efficiently on resource-constrained devices without compromising performance is a significant challenge. Implementing federated learning protocols for edge devices introduces additional complexities related to communication, synchronization, and security. Designing robust federated learning frameworks that can effectively train AI models across distributed devices while preserving data privacy and security requires careful consideration and expertise.

Most studies included in this review focused on the application of wearable AI for the detection of existing sleep apnea, its type, or its severity, rather than the anticipation of its occurrence. Foreseeing the onset of sleep apnea in the future is as pivotal as, if not more pivotal than, recognizing the current sleep apnea state, as it can pave the way for the development and implementation of proactive interventions. Consequently, we encourage researchers to undertake additional investigations into the capacity of wearable AI to predict future instances of sleep apnea. Such studies collect longitudinal data over an extended period to train and validate predictive models accurately. However, obtaining continuous and comprehensive sleep data from individuals over time can be challenging due to factors such as participant compliance, dropout rates, and the need for long-term monitoring.

In this review, only a single study evaluated the effectiveness of wearable AI in identifying CSA. In addition, only 7 (18%) of the 38 studies investigated the capability of wearable AI to differentiate between different types of sleep apnea. More research is urgently needed to evaluate the performance of wearable AI in these crucial areas. Our study also suggests that more open-source data sets with prepared manual labels for different types of sleep apnea are needed. Collecting large-scale, comprehensive, and well-annotated data from individuals with CSA poses challenges due to the rarity of CSA cases. Furthermore, identifying informative features and physiological signals from wearable devices that can distinguish between different types of sleep apnea is challenging due to the overlap in the clinical presentation and physiological characteristics of different types of sleep apnea, particularly in cases of mixed sleep apnea where both obstructive and central events occur concurrently.

Merely 3 (8%) of the 38 included studies used self-reported data and nonwearable device data alongside wearable device data for the detection of sleep apnea. The inclusion of self-reported data (eg, data regarding demographics, BMI, medical history, family history, and medications) and nonwearable device data (eg, data collected via mobile phones, smart pillows, smart mattresses, voice recorders, and Internet of Things) has the potential to enhance the efficacy of wearable AI in identifying sleep apnea. Hence, manufacturers and researchers are encouraged to take these types of data into consideration, alongside wearable device data, when developing wearable AI for the diagnosis of sleep apnea. However, challenges arise in transferring nonwearable data to wearable devices and the potential impact on the performance of wearable devices in terms of processing speed, memory use, energy consumption, synchronization, and security.

A few studies in this review compared the performance of wearable devices worn on different parts of the body (eg, wrist, abdomen, and chest) and developed wearable AI for not only detecting but also intervening in sleep apnea. This points to a crucial gap in research, which urges further investigation into the different performances of wearable AI with different placements and integrated treatment delivery via wearable AI for sleep apnea management.

Among the 38 studies in our review, 11 (29%) were excluded from the meta-analyses due to insufficient details crucial for their conduct (eg, confusion matrices and the number of apnea and nonapnea cases). They also did not provide multiple performance measures (eg, accuracy, sensitivity, and specificity), which are essential for estimating the necessary information. It is recommended that researchers include these specific details in their reports to facilitate the conduct of meta-analyses by other researchers. However, we acknowledge that the space constraints imposed by journals and conference proceedings may present a challenge for researchers seeking to include more comprehensive details in their reports.

### Limitations

Our review intentionally excluded studies involving (1) nonwearable devices, near-body wearable devices, in-body wearable devices, wearable devices wired to nonwearable devices, and wearable devices requiring an expert for their placement on users; (2) wearable AI in detecting other sleep disorders (eg, insomnia, narcolepsy, and restless legs syndrome); and (3) wearable AI in predicting outcomes of sleep apnea interventions or detecting sleep quality or sleep stages. Therefore, our findings are specifically applicable to wearable AI for sleep apnea detection and may not be generalizable to the excluded devices, disorders, or outcomes. Our findings are based on studies conducted in only 16 countries. Further, while most studies were carried out in hospitals, only 4 (11%) of the 38 studies were conducted in health care centers. Therefore, extrapolating our results to broader populations and clinical settings requires caution. This limitation acknowledges the need for further reviews in these broader areas.

Another limitation of this review is the likelihood of an overestimation or underestimation of the results of our meta-analyses due to 2 reasons. First, some relevant studies could have been overlooked, as our search was confined to English-language publications, and we did not explore other widely used databases, such as CINAHL and Web of Science. Secondly, 11 of the 38 studies in this review were excluded from the meta-analyses, as they did not report details required for meta-analyses.

### Conclusions

Our review underscores the potential of wearable AI in identifying sleep apnea, differentiating its type, and gauging its severity. However, wearable AI is not yet ready for integration into routine clinical practices due to its suboptimal performance. Therefore, until further evidence demonstrates an ideal performance, we suggest the concurrent use of wearable AI with traditional sleep apnea assessments (eg, polysomnography and home sleep apnea testing), rather than a complete substitution. Manufacturers need to develop certified commercial wearable devices that can easily detect sleep apnea, predict its occurrence, and deliver proactive interventions. CNN in particular or deep learning algorithms in general should be prioritized during the development of wearable AI for the detection of sleep apnea. Further studies are needed to assess the performance of wearable AI in detecting CSA and distinguishing it from other types of sleep apnea. Researchers should consider incorporating self-reported and nonwearable device data alongside wearable data to enhance the efficacy of wearable AI in detecting sleep apnea. Additional research is required to evaluate the varying performance of wearable devices with different placements. Researchers should also report sufficient details about their findings to enable other researchers to conduct meta-analyses effectively.

## Appendix

Multimedia Appendix 1 PRISMA-DTA (Preferred Reporting Items for Systematic Reviews and Meta-Analyses extension for Diagnostic Test Accuracy) checklist.

Multimedia Appendix 2 Search strategy.

Multimedia Appendix 3 Data extraction form.

Multimedia Appendix 4 Modified version of Quality Assessment of Studies of Diagnostic Accuracy-Revised.

Multimedia Appendix 5 Characteristics of each included study.

Multimedia Appendix 6 Features of wearable devices.

Multimedia Appendix 7 Features of artificial intelligence algorithms.

Multimedia Appendix 8 Reviewers’ judgments about each “risk of bias” and applicability domain for each included study.

## Abbreviations

AI: artificial intelligence
CNN: convolutional neural network
CSA: central sleep apnea
HR: heart rate
ML: machine learning
OSA: obstructive sleep apnea
PRISMA-DTA: Preferred Reporting Items for Systematic Reviews and Meta-Analyses extension for Diagnostic Test Accuracy
QUADAS-2: Quality Assessment of Studies of Diagnostic Accuracy-Revised

## Data Availability

The data that support the findings of this study are available from the corresponding author upon reasonable request.
