# Supplementary material for: Detection of Sleep Apnea Using Wearable AI: Systematic Review and Meta-Analysis
Source: J Med Internet Res. 2024 Sep 10;26:e58187. doi: 10.2196/58187 (PMC11422752; doi:10.2196/58187)
Supplement: Multimedia Appendix 3 [file jmir_v26i1e58187_app3.docx]

| **Extracted data** | **Definition** |
| --- | --- |
| **Study Characteristics** |  |
| Author | The first author of the study (2nd name). |
| Year of publication | The year in which the study was published. |
| Country of publication | The country where the study was conducted (where was the data collected). |
| Type of publication | The venue where the study was published: peer-reviewed journal articles, conference proceedings, or dissertations. |
| **Participants Characteristics** |  |
| Number of participants | What is the number of participants from which the data was collected? |
| Mean age | What is the mean age of the participants in the study? |
| Age range | What is the age range of participants in the study? |
| Female percentage | What is the female percentage of the participants? |
| Mean BMI | What is mean BMI of the participants in the study? |
| Type of sleep apnea | Which type of sleep apnea the study focused on (Obstructive sleep apnea (OSA) and Central sleep apnea (CSA), Mixed apnea (MA), hypopnea (HYPO))? |
| **Wearable devices characteristics** |  |
| Status of the wearable device | Is the wearable device a prototype (non-commercial) or is it an already available commercial device (e.g., Fitbit, apple watch)? |
| Name of the wearable device | What is the name of the wearable device (e.g., Fitbit, Empatica, ApplyWatch, ActiWatch, etc..)? |
| Placement of the wearable device | Where the wearable device is worn during the experiment in paper or normally (wrist, chest, head, ears, forehead, eyes, fingers, foot, etc..)? |
| Duration of wearing the WD | How long was the wearable device worn by participants? |
| **AI** **Characteristics** |  |
| Problem solving approaches | What is the problem-solving approach that the algorithm follows (Classification, regression, clustering)? |
| AI algorithm used | What are the AI algorithms/models (e.g., RF, SVM, ANN, CNN, RNN, DNN, k-NN, MLP, DBN, DBM, DPN BN, CRT, DT, LASSO, LR, MFA, MLR, MDL, NB, NN, NSC, RBFN) used in the paper? |
| Aim of AI algorithm | What was the aim of the algorithm: Detection of current current/past state OR Prediction of future state? |
| Dataset size | What is data samples, records, data points that were collected from participants to develop the model? |
| Data sources | What is the source of data that was used for developing the algorithms (open source or closed source)? |
| Data types | What is the type of data (e.g., WD-based data, self-reported data, non-WD-based data) that was used for developing the algorithm? |
| Data input to AI algorithm | What is the data that was used for developing the algorithm (heart rate, respiration rate, ECG, body movement, etc.)? |
| Number of features | How many features of the collected data are used to train AI model. |
| Type of validation | What is the approach that was used to validate the developed algorithm (e.g., Training-test split, K-fold cross-validation, Nested Cross-Validation, Leave One Out cross-validation, Apparent validation, external validation)? |
| **Ground truth & Outcomes** |  |
| Ground truth assessment (Reference standard) | How the actual status (e.g., diagnosis) of the user was confirmed (questionnaires, PSG)? |
| Guidelines used | What are the guidelines used to identify the ground truth (e.g., AASM)? |
| Assessor of the ground truth | Who did label/identify the ground truth? |
| Measured outcomes | What are the measured outcomes that the study assessed (detecting patients with sleep apnea (apnea vs. no apnea), sleep apnea severity detection (mild, moderate, severe), apnea events in respiration (apnea vs. no apnea), and type of apnea events in respiration (OSA, CSA, HYP))? |
| Results | What is the highest accuracy, sensitivity, specificity, r for each algorithm for each outcome measure? |
