# Supplementary material for: Detection of Sleep Apnea Using Wearable AI: Systematic Review and Meta-Analysis
Source: J Med Internet Res. 2024 Sep 10;26:e58187. doi: 10.2196/58187 (PMC11422752; doi:10.2196/58187)
Supplement: Multimedia Appendix 4 [file jmir_v26i1e58187_app4.docx]

Appendix 4: The modified version of QUADAS-2

| **1. Participants** | **Signaling questions** | **Explanation** |
| --- | --- | --- |
|  | 1.1 Was a consecutive or random sample of patients enrolled? | -**Yes**: if a consecutive or random sample of eligible patients was enrolled.  - **No**: if patients were selected by convenience;  - **Unclear**: if the study did not report the manner in which participants were enrolled. |
|  | 1.2 Did the study avoid inappropriate exclusions? | - **Yes**: If inclusion and exclusion of participants were appropriate, so participants correspond to unselected participants of interest.  - **No**: If participants are included who would already have been identified as having the outcome and so are no longer participants at the suspicion of disease (diagnostic studies),  or if specific subgroups are excluded that may have altered the performance of the prediction model for the intended target population.  - **Unclear**: When there is no information on whether inappropriate inclusions or exclusions took place. |
|  | 1.3 Was the sample size sufficient? | - **Yes**: either having a minimum of 100 participants or in studies where multiple samples are recorded for each participant, having at least 100 samples. The latter condition is applicable when the input to the machine learning algorithm is derived from individual samples, rather than from entire subjects.  - **No**: if there are less than 100 participants, or in studies where multiple samples are recorded for each participant, if there are fewer than 100 samples.  - **Unclear**: no information on the number of features or number of participants. |
|  | 1.4 Was there a balance in the number of patients between the subgroups? | - **Yes**: if the percentage of participants in any group is 66.7% or less of the total sample (≤2/3).  - **No**: if the percentage of participants in any group is more than 66.7% of the total sample (>2/3).  - **Unclear**: If no information was provided regarding the number of participants in the groups. |
|  | **Risk-of-bias assessment:** Could the selection of participants have introduced bias? | - **Low risk of bias**: If the answer to all signaling questions is ‘Yes’ then the risk of bias can be considered low. If one or more of the answers is ‘No’, the judgment could still be low risk of bias, but specific reasons why the risk of bias can be considered low should be provided.  - **High risk of bias**: If the answer to any of the signaling questions is “No” there is a potential for bias, except if defined at low risk of bias above.  - **Unclear risk of bias**: If relevant information is missing for all or some of the signaling questions, and none of the answers to signaling questions is judged to put this domain at high risk of bias. |
|  | **Concerns regarding applicability:** Are there concerns that the included participants and setting do not match the review question? | - **Low concern for applicability**: If the spectrum of participants (in- and exclusion criteria, setting, prior testing) matches the pre-stated requirements in the review question  - **High concern for applicability**: If the spectrum of participants does not fully match the pre-stated requirements in the review question  - **Unclear concern for applicability**: If there is insufficient information available to make a judgment about the applicability |
| **2. Index test (AI algorithms)** | 2.1 Were the AI models described in detail? | -**Yes**: if the model details were provided such as outputs, epochs, number of layers, pooling, normalization, regularization, and activation in the layers, etc.  or if a previously published model is  employed, the paper must cite a reference that meets the preceding standards and fully describe every modification made to the model.  -**No**: if only the model’s name was reported in the paper, or the study reported some information but other important information still missing. |
|  | 2.2 Were all features (predictors) used in the model clearly identified? | -**Yes**: If all features used in each model were clearly reported.  -**No**: If any features used in any model were not reported.  Or all features used in the paper were identified but it was not clear which features were used with each model. |
|  | 2.3 Were features assessed in the same way for all participants? | Please notice some studies used different wearable devices to collect the data.  -**Yes**: If the assessment of features was similar for all participants.  -**No**: If different definitions were used for the same predictor or if predictors requiring subjective interpretation were assessed by differently experienced assessors.  **-Unclear**: If there is no information on how predictors were defined or assessed. |
|  | 2.4 Were features collected without knowledge of outcome data (sleep apnea)? | - **Yes**: If outcome information was stated as not used during feature assessment or was clearly not (yet) available to those assessing features.  - **No**: If it is clear that outcome information was used when assessing predictors.  - **Unclear**: No information on whether features were assessed without knowledge of outcome information. |
|  | **Risk-of-bias assessment:** Could the conduct or interpretation of the index test have introduced bias? | - **Low risk of bias**: If the answer to all signaling questions is ‘Yes’ then the risk of bias can be considered low. If one or more of the answers is ‘No’, the judgment could still be low risk of bias, but specific reasons why the risk of bias can be considered low should be provided e.g., the use of objective predictors not requiring subjective interpretation.  - **High risk of bias**: If the answer to any of the signaling questions is “No” there is a potential for bias, except if defined at low risk of bias above.  - **Unclear risk of bias**: If relevant information is missing for all or some of the signaling questions, and none of the answers to signaling questions is judged to put this domain at high risk of bias. |
|  | **Concerns regarding applicability:** Are there concerns that the definition, assessment, or timing of the index test in the model does not match the review question? | - **Low concern for applicability**: Definition, assessment, and timing of predictors match the review question.  - **High concern for applicability**: Definition, assessment, or timing of predictors were different from the review question.  - **Unclear concern for applicability**: If relevant information about the predictors is not reported. |
| **3. Reference Standard (Ground truth)** | 3.1 Was the reference standard likely to correctly classify the outcome (e.g., "sleep apnea" or "non-sleep apnea")? | Is the used tool appropriate?  Were the assessors/annotators qualified?  **Yes**: If the study:   - Followed the American Academy of Sleep Medicine (AASM) guidelines to detect sleep apnea/respiratory events from data collected by Polysomnography (PSG), Home Sleep Apnea Testing (HSAT), or other relevant tests. - Appropriately used unsupervised learning to generate labels. - Used an appropriate experimental setup to assess sleep apnea and/or respiratory events.   -**No**: If an unknown reference test, with unverified validity and reliability, was used, or if the sleep monitoring was conducted by unqualified assessors.  - **Unclear**: If no information was provided about the reference standard. |
|  | 3.2 Was the outcome defined and determined in a similar way for all participants? | - **Yes**: If:  - outcomes were defined and determined in a similar way for all participants.  - The same duration for the diagnostic test was applied consistently across all study participants.  - **No**:  - If outcomes were clearly defined and determined in a different way for some participants.  - Different duration for the diagnostic test was applied across study participants.  - **Unclear**: No information on whether outcomes were defined or determined in a similar way for all participants. |
|  | 3.3 Was the outcome determined without knowledge of predictor information? | - **Yes**: If predictor information was not known when determining the outcome status, or outcome status determination is clearly reported as determined without knowledge of predictor information.  - **No**: If it is clear that predictor information was used when determining the outcome status.  - **Unclear**: No information on whether the outcome was determined without knowledge of predictor information. |
|  | 3.4  Was the diagnostic test conducted for an appropriate duration to ensure accurate results? | **Yes, if:**   - The test duration is consistent with established guidelines for sleep apnea diagnosis, which is > 4 hrs for the PSG test.   **No,** If:   - The test duration is not consistent with established guidelines for sleep apnea diagnosis. - OR the duration was not long enough to capture a representative sample of the patient's sleep data.   - **Unclear**: If no information was provided on the duration of the diagnostic test or the experiment. |
|  | **Risk-of-bias assessment:** Could the reference standard, its conduct, or its interpretation have introduced bias? | - **Low risk of bias**: If the answer to all signaling questions is ‘Yes’ then the risk of bias can be considered low. If one or more of the answers is ‘No’, the judgment could still be low risk of bias, but specific reasons why the risk of bias can be considered low should be provided e.g., when the outcome was  determined with knowledge of predictor information but the outcome assessment did not require much interpretation by the assessor (e.g., death regardless of cause).  - **High risk of bias**: If the answer to any of the signaling questions is “No” there is a potential for bias, except if defined at low risk of bias above.  - **Unclear risk of bias**: If relevant information is missing for all or some of the signaling questions, and none of the answers to signaling questions is judged to put this domain at high risk of bias. |
|  | **Concerns regarding applicability:** Are there concerns that the outcome definition, timing, or determination do not  match the review question? | - **Low concern for applicability**: Outcome definition, timing, and method of determination define the outcome as intended by the review question.  -**High concern for applicability**: Choice of outcome definition, timing, and method of outcome determination defines another outcome as intended by the review question.  - **Unclear concern for applicability**: If relevant information about the outcome, timing, and method of determination is not reported. |
| **4.** **Analysis** | 4.1 Were all participants included in the analysis? | - **Yes**: If all participants enrolled in the study are  included in the data analysis.  - **No**: If some or a subgroup of participants are  inappropriately excluded from the analysis.  - **Unclear**: No information on whether all enrolled participants are included in the analysis. |
|  | 4.2 Was data preprocessing carried out appropriately? | - **Yes**: If there are no missing values of predictors or outcomes and the study explicitly reports that participants are not excluded on the basis of missing data, or if missing values are handled using multiple imputation.  - **No**: If participants with missing data are omitted from the analysis, or if the method of handling missing data is clearly flawed, e.g., missing indicator method or inappropriate use of last value carried forward, or if the study had no explicit mention of methods to handle missing data.  - **Unclear**: If there is insufficient information to determine if the method of handling missing data is appropriate. |
|  | 4.3 Was the breakdown of the training, validation, and test sets appropriate? | - **Yes**:   - If there's a clear rationale for the chosen distribution and it aligns with best practices in the field (e.g., 70% training, 15% validation, 15% test).   - **No**:   - If the sizes of the validation and test sets are too small to reliably evaluate the model's performance. - If there’s a lack of justification or clear rationale for how the sets were divided, or if the division contradicts standard practices without a valid reason.   - **Unclear**: If the study does not provide specific information on how the training, validation, and test sets were divided. |
|  | 4.4 Was the performance of the model evaluated appropriately? | - **Yes**: If the confusion matrix was presented,  Or more than one measure was used and the selected measures were appropriate.  - **No**: If the confusion matrix was not presented, and only one measure was reported,  Or the selected measures were not appropriate.  - **Unclear**: If no information was provided on the performance measures |
|  | **Risk-of-bias assessment:** Could the analysis, its conduct, or its interpretation have introduced bias? | - **Low risk of bias**: If the answer to all signaling questions is ‘Yes’ then the risk of bias can be considered low. If one or more of the answers is ‘No’, the judgment could still be low risk of bias, but specific reasons why the risk of bias can be considered low should be provided.  - **High risk of bias:** If the answer to any of the signaling questions is “No” there is a potential for bias, except if defined at low risk of bias above.  - **Unclear risk of bias**: If relevant information is missing for all or some of the signaling questions, and none of the answers to signaling questions is judged to put the analysis at high risk of bias. |
