# Supplementary material for: Detection of Sleep Apnea Using Wearable AI: Systematic Review and Meta-Analysis
Source: J Med Internet Res. 2024 Sep 10;26:e58187. doi: 10.2196/58187 (PMC11422752; doi:10.2196/58187)
Supplement: Multimedia Appendix 5 [file jmir_v26i1e58187_app5.docx]

**Multimedia Appendix 5: Characteristics of each Included Study**

| Study [Ref] | Year | Publication type | Country | Sample size | Mean age | Age range | Female % | Sleep apnea Type | |
| --- | --- | --- | --- | --- | --- | --- | --- | --- | --- |
| Benedetti [1] | 2022 | Journal article | Italy | 78 | 57.2 | NR | 38.5 | | OSA |
| Chang [2] | 2020 | Journal article | Taiwan | 115 | 48.08 | NR | 21.7 | | All |
| Chen M [3] | 2022 | Journal article | China | 92 | NR | NR | NR | | OSA |
| Chen X [4] | 2021 | Conference paper | United States | 20 | 59.3 | 36-72 | 60 | | All |
| Fallmann [5] | 2018 | Conference paper | United States | 2252 | NR | NR | NR | | OSA |
| Fedorin [6] | 2020 | Conference paper | South Korea | 165 | 39.6 | NR | NR | | All |
| Ganglberger [7] | 2022 | Journal article | United States | 409 | 56 | NR | 46 | | All |
| Gu [8] | 2020 | Journal article | United States | 50 | 54.9 | NR | 46 | | OSA |
| Hafezi [9] | 2019 | Conference paper | Canada | 20 | 48.2 | NR | 45 | | All |
| Hafezi [10] | 2020 | Journal article | Canada | 69 | 52.1 | NR | 46.4 | | All |
| Hung [11] | 2018 | Conference paper | Vietnam | 5 | NR | NR | NR | | CSA |
| Jeon [12] | 2020 | Conference paper | South Korea | 8 | NR | NR | NR | | All |
| Ji [13] | 2022 | Journal article | Singapore | 18 | NR | NR | 50 | | All |
| Kristiansen[14] | 2021 | Journal article | Norway | 579 | 59.9 | 20-39 | 27.1 | | All |
| Kristiansen [15] | 2023 | Journal article | Norway | 29 | 61.1 | NR | 27.6 | | All |
| Kwon [16] | 2023 | Journal article | United States | 48 | NR | NR | NR | | All |
| Le [17] | 2013 | Journal article | United States | 8 | NR | NR | NR | | OSA |
| McClure [18] | 2020 | Journal article | United States | 100 | 33.2 | NR | 54 | | All |
| Papini [19] | 2020 | Journal article | Netherlands | 502 | 48 | 18-82 | 37.1 | | OSA |
| Petrenko [20] | 2020 | Conference paper | Ukraine | 8 | 36.25 | 25-55 | 50 | | All |
| Rossi [21] | 2023 | Journal article | Italy | 20 | 45 | 24-67 | 20 | | All |
| Ryser [22] | 2022 | Journal article | Switzerland | 13 | 25.6 | 19-33 | 46.2 | | All |
| Selvaraj [23] | 2014 | Conference paper | India | 53 | 42.3 | 22-73 | 45.3 | | All |
| Shen [24] | 2022 | Journal article | China | 92 | NR | NR | NR | | All |
| Strumpf [25] | 2023 | Journal article | United States | 84 | 48.3 | NR | 50 | | OSA |
| Tsouti [26] | 2020 | Journal article | Greece | 12 | NR | NR | NR | | OSA |
| Van Steenkiste [27] | 2020 | Journal article | Belgium | 25 | 57.8 | NR | 12 | | All |
| Wang [28] | 2023 | Journal article | China | 10 | 37.2 | 23-54 | 40 | | All |
| Wang [29] | 2022 | Journal article | China | 62 | NR | 24-77 | 21 | | OSA |
| Wu [30] | 2018 | Journal article | Taiwan | 62 | 46.9 | NR | 21 | | All |
| Wu [31] | 2021 | Journal article | China | 92 | NR | NR | NR | | All |
| Xu [32] | 2023 | Journal article | China | 196 | 45.1 | 18-80 | 14 | | OSA |
| Yeh [33] | 2021 | Journal article | United States | 78 | 51.5 | NR | 65 | | OSA |
| Yeo [34] | 2022 | Journal article | South Korea | 96 | 48 | NR | 26 | | All |
| Yeo [35] | 2022 | Journal article | South Korea | 94 | 48 | NR | NR | | All |
| Yüzer [36] | 2020 | Journal article | Turkey | 5 | NR | NR | NR | | All |
| Zhang [37] | 2023 | Journal article | China | 4 | 32.8 | 27-42 | 25 | | All |
| Zhou [38] | 2023 | Conference paper | China | 350 | NR | NR | NR | | OSA |
| NR: Not reported, OSA: Obstructive sleep apnea | | | | | | | | | |

1. Benedetti, D., et al., *Obstructive Sleep Apnoea Syndrome Screening Through Wrist-Worn Smartbands: A Machine-Learning Approach.* Nat Sci Sleep, 2022. **14**: p. 941-956.

2. Chang, H.C., et al., *Portable Sleep Apnea Syndrome Screening and Event Detection Using Long Short-Term Memory Recurrent Neural Network.* Sensors (Basel), 2020. **20**(21).

3. Chen, M., et al., *Information-Based Similarity of Ordinal Pattern Sequences as a Novel Descriptor in Obstructive Sleep Apnea Screening Based on Wearable Photoplethysmography Bracelets.* Biosensors (Basel), 2022. **12**(12).

4. Chen, X., et al., *ApneaDetector: Detecting Sleep Apnea with Smartwatches.* Proc. ACM Interact. Mob. Wearable Ubiquitous Technol., 2021. **5**(2): p. Article 59.

5. Fallmann, S. and L. Chen. *Detecting Chronic Diseases from Sleep-Wake Behaviour and Clinical Features*. in *2018 5th International Conference on Systems and Informatics (ICSAI)*. 2018.

6. Fedorin, I., K. Slyusarenko, and M. Nastenko, *Respiratory events screening using consumer smartwatches*. 2020. 25-28.

7. Ganglberger, W., et al., *Sleep apnea and respiratory anomaly detection from a wearable band and oxygen saturation.* Sleep Breath, 2022. **26**(3): p. 1033-1044.

8. Gu, W., et al., *Belun Ring Platform: a novel home sleep apnea testing system for assessment of obstructive sleep apnea.* J Clin Sleep Med, 2020. **16**(9): p. 1611-1617.

9. Hafezi, M., et al., *Sleep Apnea Severity Estimation From Tracheal Movements Using a Deep Learning Model.* IEEE Access, 2020. **8**: p. 22641-22649.

10. Hafezi, M., et al. *Sleep Apnea Severity Estimation from Respiratory Related Movements Using Deep Learning*. in *2019 41st Annual International Conference of the IEEE Engineering in Medicine and Biology Society (EMBC)*. 2019.

11. Hung, P.D., *Central Sleep Apnea Detection Using an Accelerometer*, in *Proceedings of the 1st International Conference on Control and Computer Vision*. 2018, Association for Computing Machinery: Singapore, Singapore. p. 106–111.

12. Jeon, Y., K. Heo, and S.J. Kang, *Real-Time Sleep Apnea Diagnosis Method Using Wearable Device without External Sensors*. 2020. 1-5.

13. Ji, X., et al., *Airline Point-of-Care System on Seat Belt for Hybrid Physiological Signal Monitoring.* Micromachines, 2022. **13**(11): p. 1880.

14. Kristiansen, S., et al., *Machine Learning for Sleep Apnea Detection with Unattended Sleep Monitoring at Home.* ACM Trans. Comput. Healthcare, 2021. **2**(2): p. Article 14.

15. Kristiansen, S., et al., *A clinical evaluation of a low-cost strain gauge respiration belt and machine learning to detect sleep apnea.* Smart Health, 2023. **27**: p. 100373.

16. Kwon, S., et al., *At-home wireless sleep monitoring patches for the clinical assessment of sleep quality and sleep apnea.* Science Advances, 2023. **9**(21): p. eadg9671.

17. Le, T.Q., et al., *Wireless Wearable Multisensory Suite and Real-Time Prediction of Obstructive Sleep Apnea Episodes.* IEEE J Transl Eng Health Med, 2013. **1**: p. 2700109.

18. McClure, K., et al., *Classification and Detection of Breathing Patterns with Wearable Sensors and Deep Learning.* Sensors (Basel), 2020. **20**(22).

19. Papini, G.B., et al., *Wearable monitoring of sleep-disordered breathing: estimation of the apnea-hypopnea index using wrist-worn reflective photoplethysmography.* Sci Rep, 2020. **10**(1): p. 13512.

20. Petrenko, A. *Breathmonitor: Sleep Apnea Mobile Detector*. in *2020 IEEE 2nd International Conference on System Analysis & Intelligent Computing (SAIC)*. 2020.

21. Rossi, M., et al., *SLEEP-SEE-THROUGH: Explainable Deep Learning for Sleep Event Detection and Quantification From Wearable Somnography.* IEEE J Biomed Health Inform, 2023. **27**(7): p. 3129-3140.

22. Ryser, F., et al., *Respiratory analysis during sleep using a chest-worn accelerometer: A machine learning approach.* Biomedical Signal Processing and Control, 2022. **78**: p. 104014.

23. Selvaraj, N. and R. Narasimhan, *Automated prediction of the apnea-hypopnea index using a wireless patch sensor.* 2014 36th Annual International Conference of the IEEE Engineering in Medicine and Biology Society, EMBC 2014, 2014. **2014**: p. 1897-900.

24. Shen, Q., et al., *Multitask Residual Shrinkage Convolutional Neural Network for Sleep Apnea Detection Based on Wearable Bracelet Photoplethysmography.* IEEE Internet of Things Journal, 2022. **9**(24): p. 25207-25222.

25. Strumpf, Z., et al., *Belun Ring (Belun Sleep System BLS-100): Deep learning-facilitated wearable enables obstructive sleep apnea detection, apnea severity categorization, and sleep stage classification in patients suspected of obstructive sleep apnea.* Sleep Health, 2023. **9**(4): p. 430-440.

26. Tsouti, V., et al., *Development of an automated system for obstructive sleep apnea treatment based on machine learning and breath effort monitoring.* Microelectronic Engineering, 2020. **231**: p. 111376.

27. Van Steenkiste, T., et al., *Portable Detection of Apnea and Hypopnea Events Using Bio-Impedance of the Chest and Deep Learning.* IEEE Journal of Biomedical and Health Informatics, 2020. **PP**: p. 1-1.

28. Wang, S., et al., *Machine Learning Assisted Wearable Wireless Device for Sleep Apnea Syndrome Diagnosis.* Biosensors, 2023. **13**(4): p. 483.

29. Wang, Z., et al., *Single-lead ECG based multiscale neural network for obstructive sleep apnea detection.* Internet of Things, 2022. **20**: p. 100613.

30. Wu, H.T., et al., *Phenotype-Based and Self-Learning Inter-Individual Sleep Apnea Screening With a Level IV-Like Monitoring System.* Front Physiol, 2018. **9**: p. 723.

31. Wu, S., et al., *Sleep apnea screening based on Photoplethysmography data from wearable bracelets using an information-based similarity approach.* Computer Methods and Programs in Biomedicine, 2021. **211**: p. 106442.

32. Xu, Y., et al., *Comparative study of a wearable intelligent sleep monitor and polysomnography monitor for the diagnosis of obstructive sleep apnea.* Sleep Breath, 2023. **27**(1): p. 205-212.

33. Yeh, E., et al., *Detection of obstructive sleep apnea using Belun Sleep Platform wearable with neural network-based algorithm and its combined use with STOP-Bang questionnaire.* PLoS One, 2021. **16**(10): p. e0258040.

34. Yeo, M., et al., *Respiratory Event Detection During Sleep Using Electrocardiogram and Respiratory Related Signals: Using Polysomnogram and Patch-Type Wearable Device Data.* IEEE J Biomed Health Inform, 2022. **26**(2): p. 550-560.

35. Yeo, M., et al., *Robust Method for Screening Sleep Apnea With Single-Lead ECG Using Deep Residual Network: Evaluation With Open Database and Patch-Type Wearable Device Data.* IEEE Journal of Biomedical and Health Informatics, 2022. **26**(11): p. 5428-5438.

36. Yüzer, A.H., et al., *A different sleep apnea classification system with neural network based on the acceleration signals.* Applied Acoustics, 2020. **163**: p. 107225.

37. Zhang, H., et al., *Long-Term Sleep Respiratory Monitoring by Dual-Channel Flexible Wearable System and Deep Learning-Aided Analysis.* IEEE Transactions on Instrumentation and Measurement, 2023. **72**: p. 1-9.

38. Zhou, G., et al., *Automatic monitoring of obstructive sleep apnea based on multi-modal signals by phone and smartwatch.* Annu Int Conf IEEE Eng Med Biol Soc, 2023. **2023**: p. 1-4.
