# Supplementary material for: Detection of Sleep Apnea Using Wearable AI: Systematic Review and Meta-Analysis
Source: J Med Internet Res. 2024 Sep 10;26:e58187. doi: 10.2196/58187 (PMC11422752; doi:10.2196/58187)
Supplement: Multimedia Appendix 7 [file jmir_v26i1e58187_app7.docx]

**Multimedia Appendix 7: Features of AI Algorithms**

| Study [Ref] | Problem solving approach | AI algorithm | Aim of AI algorithm | Dataset size | Dataset source | Data type | Data input | Number of features | Ground Truth Assessment | Guidelines | Assessor | Type of Validation | Measured Outcome |
| --- | --- | --- | --- | --- | --- | --- | --- | --- | --- | --- | --- | --- | --- |
| Benedetti [1] | Classification | MLP, RF | Detection | 78 | Closed source | WD-based | Body movement, heart rate data | 11 | WD | AASM | Sleep technicians | LOOCV | SA detection, SA severity |
| Chang [2] | Classification | LSTM | Detection | NR | Closed source | WD-based | Oxygen saturation, respiration data | 9 | PSG | AASM | Sleep technicians | Training-test split | RE detection, RE type, SA detection, SA severity |
| Chen M [3] | Classification | DT, KNN, NB, RF | Detection | 7619 | Closed source | WD-based | Heart rate data | 10 | PSG | AASM | Sleep technicians | K-fold CV | SA severity |
| Chen X [4] | Classification | AB, DT, NB, RF, SVM | Detection | 4961 | Closed source | WD-based | Respiration data | 5 | PSG | AASM | Sleep physicians | K-fold CV | RE detection, SA detection, SA severity |
| Fallmann [5] | Classification | LSTM | Detection, prediction | 2252 | Open source | WD-based, self-reported | NR | NR | WD | AASM | Sleep technicians | K-fold CV | RE detection, SA detection |
| Fedorin [6] | Classification, Regression | LSTM | Detection | NR | Closed source | WD-based | Body movement, heart rate data, respiration data | 190 | PSG | AASM | Sleep technicians | Training-test split | RE detection, SA severity |
| Ganglberger [7] | Classification, Regression | RF | Detection | 561480 | Closed source | WD-based | Oxygen saturation, respiration data | 10 | PSG | AASM | Sleep technicians | K-fold CV | RE detection, RE type, SA detection, SA severity |
| Gu [8] | Classification, Regression | ANN | Detection | 8476 | Closed source | WD-based | Body movement, heart rate data, oxygen saturation | 74 | PSG | AASM | Sleep technicians | Training-test split | SA detection, SA severity |
| Hafezi [9] | Classification, Regression | CNN, LSTM | Detection | 20 | Closed source | WD-based | Respiration data | 21 | PSG | AASM | NA | K-fold CV | RE detection |
| Hafezi [10] | Classification, Regression | CNN, LSTM | Detection | 69 | Closed source | WD-based | Respiration data | 21 | PSG | AASM | NR | K-fold CV | RE detection |
| Hung [11] | Classification | MLP | Detection | NR | Closed source | WD-based | Heart rate data, respiration data | 13 | Context | NA | NA | K-fold CV | RE detection |
| Jeon [12] | Classification | ANN, KNN, NB | Detection | NR | Closed source | WD-based | Body movement, heart rate data | NR | WD | AASM | NR | K-fold CV, training-test split | RE detection |
| Ji [13] | Classification | AB, BP, DT, KNN, LSTM, NB, QDA, RF, SVM | Detection | 3240 | Closed source | WD-based | Body movement, heart rate data, respiration data | 3 | Context | NA | NA | Training-test split | RE detection |
| Kristiansen [14] | Classification | CNN, GRU, KNN, LSTM, MLP, RF, SVM | Detection | 228018 | Closed source | WD-based | Oxygen saturation, respiration data | NR | WD | AASM | Sleep technicians | K-fold CV | RE detection, RE type |
| Kristiansen [15] | Classification, Regression | CNN, GRU, LSTM, MLP, RF | Detection | 13105 | Closed source | WD-based | Oxygen saturation, respiration data | NR | WD | AASM | Sleep technicians | K-fold CV, LOOCV | RE detection, SA detection, SA severity |
| Kwon [16] | Classification | CNN | Detection | 40883 | Closed source | WD-based | EEG, EOG, EMG | NR | PSG | AASM | Sleep technicians | Training-test split | RE detection |
| Le [17] | Classification, Regression | SVM | Prediction | NR | Closed source | WD-based | Acoustic data, heart rate data, oxygen saturation, respiration data | 14 | WD | NR | Sleep technicians | K-fold CV | RE detection |
| McClure [18] | Classification | CNN | Detection | NR | Closed source | WD-based | Respiration data | 12 | Context | NA | Sleep physicians | Training-test split | RE detection, RE type |
| Papini [19] | Classification, Regression | CNN | Detection | 62543 | Closed source | WD-based | Body movement, features coverage, heart rate data, respiration data, sleep stage | 212 | PSG | AASM | Sleep technicians | Training-test split | RE detection, RE type, SA detection, SA severity |
| Petrenko [20] | Classification | CNN | Detection | 432000 | Closed source | WD-based | Respiration data | NR | Context | NA | NA | Training-test split | RE detection |
| Rossi [21] | Classification | CNN | Detection | 40800 | Closed source | WD-based | Acoustic data, body movement, oxygen saturation, respiration data | NR | WD | NR | Sleep technicians | LOOCV, training-test split | RE type |
| Ryser [22] | Classification | AB, KNN | Detection | 2389 | Closed source | WD-based | Respiration data | 16 | PSG | AASM | Sleep technicians | K-fold CV | RE detection |
| Selvaraj [23] | Classification, Regression | SVM | Detection | 53 | Closed source | WD-based | Body movement, heart rate data, respiration data | NR | PSG | AASM | Sleep technicians | LOOCV | SA severity |
| Shen [24] | Classification | CNN | Detection | 46124 | Closed source | WD-based | Heart rate data | NR | PSG | AASM | Sleep physicians | K-fold CV, training-test split | RE detection, SA detection |
| Strumpf [25] | Classification, Regression | CNN | Detection | 8518 | Closed source | WD-based | Body movement, heart rate data, oxygen saturation | 84 | PSG | AASM | Sleep technicians, sleep physicians | Training-test split | SA detection, SA severity |
| Tsouti [26] | Classification | ANN | Detection, prediction | 12 | Open source | WD-based | Respiration data | 3 | PSG | AASM | Sleep technicians | Training-test split | SA severity |
| Van Steenkiste [27] | Classification | LSTM | Detection | 119063 | Closed source | WD-based | Body movement, heart rate data, respiration data | NR | PSG | AASM | Sleep technicians | K-fold CV | RE detection, RE type |
| Wang [28] | Classification | KNN, RF, SVM, XGBoost | Detection | NR | Closed source | WD-based | Heart rate data, oxygen saturation | NR | PSG | AASM | Sleep physicians | NR | RE detection |
| Wang [29] | Classification | CNN | Detection | 27488 | Closed source | WD-based | Heart rate data | NR | PSG | AASM | NR | K-fold CV, training-test split | RE detection |
| Wu [30] | Classification | SVM | Detection | NR | Closed source | WD-based, non-WD based, self reported | Oxygen saturation, respiration data | 10 | PSG | AASM | Sleep technicians | LOOCV | SA detection, SA severity |
| Wu [31] | Classification, Regression | DT, KNN, NB, RF | Detection | NR | Closed source | WD-based | Heart rate data | 4 | PSG | AASM | NA | K-fold CV | SA severity |
| Xu [32] | Classification, Regression | NR | Detection | NR | Closed source | WD-based | Body movement, heart rate data, oxygen saturation | NR | PSG | AASM | Sleep physicians | Training-test split | SA detection, SA severity |
| Yeh [33] | Classification | ANN | Detection | 8495 | Closed source | WD-based | Body movement, heart rate data, oxygen saturation | 74 | PSG | AASM | Sleep technicians, sleep physicians | Training-test split | SA detection, SA severity |
| Yeo [34] | Classification, Regression | LDA, MLP, QDA, RF, SVM | Detection | 21622 | Closed source | WD-based | Heart rate data, respiration data | 135 | PSG | AASM | Sleep technicians, sleep physicians | Training-test split | RE detection, SA severity detection |
| Yeo [35] | Classification, Regression | CNN | Detection | 32004 | Closed source | WD-based | Heart rate data, respiration data | NR | PSG | AASM | Sleep technicians | Training-test split | RE detection, SA severity detection |
| Yüzer [36] | Classification | ANN | Detection | 22936 | Closed source | WD-based | Respiration data | NR | WD | AASM | NR | Training-test split | RE detection |
| Zhang [37] | Classification | CNN | Detection | 600 | Closed source | WD-based | Respiration data | NR | PSG | AASM | NR | Training-test split | RE detection, RE type |
| Zhou [38] | Classification, Regression | XGBoost | Detection | 670 | Closed source | WD-based, non-WD based, self reported | Acoustic data, body movement, demographic data, heart rate data, oxygen saturation, respiration data, risk factors | NR | PSG | AASM | Sleep technicians | K-fold CV | SA detection, SA severity |
| AASM: American Academy of Sleep Medicine; AB: AdaBoost; ANN: Artificial Neural Network; BAG: Bagging classifiers; BayesNet: Bayes network; CNN: Convolutional Neural Network; DT: Decision tree, EDA: Electrodermal activity; EEG: Electroencephalogram; EMG: Electromyography; EOG: electrooculography; KNN: K-Nearest Neighbors; LDA: Linear Discriminant Analysis; LOOCV: Leave-One-Out Cross-Validation; LSTM: Long Short-Term Memory; MLP: Multilayer perceptron; NA: Not applicable; NB: Naive Bayes; NR: Not reported; PSG: Polysomnography; QDA: Quadratic Discriminant Analysis; RE: respiratory events; RF: Random Forest; SA: sleep apnea; SVM: Support Vector Machine; SVR: Support Vector regressor; WD: Wearable device; XGBoost: extreme gradient boosting | | | | | | | | | | | | | |

1. Benedetti, D., et al., *Obstructive Sleep Apnoea Syndrome Screening Through Wrist-Worn Smartbands: A Machine-Learning Approach.* Nat Sci Sleep, 2022. **14**: p. 941-956.

2. Chang, H.C., et al., *Portable Sleep Apnea Syndrome Screening and Event Detection Using Long Short-Term Memory Recurrent Neural Network.* Sensors (Basel), 2020. **20**(21).

3. Chen, M., et al., *Information-Based Similarity of Ordinal Pattern Sequences as a Novel Descriptor in Obstructive Sleep Apnea Screening Based on Wearable Photoplethysmography Bracelets.* Biosensors (Basel), 2022. **12**(12).

4. Chen, X., et al., *ApneaDetector: Detecting Sleep Apnea with Smartwatches.* Proc. ACM Interact. Mob. Wearable Ubiquitous Technol., 2021. **5**(2): p. Article 59.

5. Fallmann, S. and L. Chen. *Detecting Chronic Diseases from Sleep-Wake Behaviour and Clinical Features*. in *2018 5th International Conference on Systems and Informatics (ICSAI)*. 2018.

6. Fedorin, I., K. Slyusarenko, and M. Nastenko, *Respiratory events screening using consumer smartwatches*. 2020. 25-28.

7. Ganglberger, W., et al., *Sleep apnea and respiratory anomaly detection from a wearable band and oxygen saturation.* Sleep Breath, 2022. **26**(3): p. 1033-1044.

8. Gu, W., et al., *Belun Ring Platform: a novel home sleep apnea testing system for assessment of obstructive sleep apnea.* J Clin Sleep Med, 2020. **16**(9): p. 1611-1617.

9. Hafezi, M., et al., *Sleep Apnea Severity Estimation From Tracheal Movements Using a Deep Learning Model.* IEEE Access, 2020. **8**: p. 22641-22649.

10. Hafezi, M., et al. *Sleep Apnea Severity Estimation from Respiratory Related Movements Using Deep Learning*. in *2019 41st Annual International Conference of the IEEE Engineering in Medicine and Biology Society (EMBC)*. 2019.

11. Hung, P.D., *Central Sleep Apnea Detection Using an Accelerometer*, in *Proceedings of the 1st International Conference on Control and Computer Vision*. 2018, Association for Computing Machinery: Singapore, Singapore. p. 106–111.

12. Jeon, Y., K. Heo, and S.J. Kang, *Real-Time Sleep Apnea Diagnosis Method Using Wearable Device without External Sensors*. 2020. 1-5.

13. Ji, X., et al., *Airline Point-of-Care System on Seat Belt for Hybrid Physiological Signal Monitoring.* Micromachines, 2022. **13**(11): p. 1880.

14. Kristiansen, S., et al., *Machine Learning for Sleep Apnea Detection with Unattended Sleep Monitoring at Home.* ACM Trans. Comput. Healthcare, 2021. **2**(2): p. Article 14.

15. Kristiansen, S., et al., *A clinical evaluation of a low-cost strain gauge respiration belt and machine learning to detect sleep apnea.* Smart Health, 2023. **27**: p. 100373.

16. Kwon, S., et al., *At-home wireless sleep monitoring patches for the clinical assessment of sleep quality and sleep apnea.* Science Advances, 2023. **9**(21): p. eadg9671.

17. Le, T.Q., et al., *Wireless Wearable Multisensory Suite and Real-Time Prediction of Obstructive Sleep Apnea Episodes.* IEEE J Transl Eng Health Med, 2013. **1**: p. 2700109.

18. McClure, K., et al., *Classification and Detection of Breathing Patterns with Wearable Sensors and Deep Learning.* Sensors (Basel), 2020. **20**(22).

19. Papini, G.B., et al., *Wearable monitoring of sleep-disordered breathing: estimation of the apnea-hypopnea index using wrist-worn reflective photoplethysmography.* Sci Rep, 2020. **10**(1): p. 13512.

20. Petrenko, A. *Breathmonitor: Sleep Apnea Mobile Detector*. in *2020 IEEE 2nd International Conference on System Analysis & Intelligent Computing (SAIC)*. 2020.

21. Rossi, M., et al., *SLEEP-SEE-THROUGH: Explainable Deep Learning for Sleep Event Detection and Quantification From Wearable Somnography.* IEEE J Biomed Health Inform, 2023. **27**(7): p. 3129-3140.

22. Ryser, F., et al., *Respiratory analysis during sleep using a chest-worn accelerometer: A machine learning approach.* Biomedical Signal Processing and Control, 2022. **78**: p. 104014.

23. Selvaraj, N. and R. Narasimhan, *Automated prediction of the apnea-hypopnea index using a wireless patch sensor.* 2014 36th Annual International Conference of the IEEE Engineering in Medicine and Biology Society, EMBC 2014, 2014. **2014**: p. 1897-900.

24. Shen, Q., et al., *Multitask Residual Shrinkage Convolutional Neural Network for Sleep Apnea Detection Based on Wearable Bracelet Photoplethysmography.* IEEE Internet of Things Journal, 2022. **9**(24): p. 25207-25222.

25. Strumpf, Z., et al., *Belun Ring (Belun Sleep System BLS-100): Deep learning-facilitated wearable enables obstructive sleep apnea detection, apnea severity categorization, and sleep stage classification in patients suspected of obstructive sleep apnea.* Sleep Health, 2023. **9**(4): p. 430-440.

26. Tsouti, V., et al., *Development of an automated system for obstructive sleep apnea treatment based on machine learning and breath effort monitoring.* Microelectronic Engineering, 2020. **231**: p. 111376.

27. Van Steenkiste, T., et al., *Portable Detection of Apnea and Hypopnea Events Using Bio-Impedance of the Chest and Deep Learning.* IEEE Journal of Biomedical and Health Informatics, 2020. **PP**: p. 1-1.

28. Wang, S., et al., *Machine Learning Assisted Wearable Wireless Device for Sleep Apnea Syndrome Diagnosis.* Biosensors, 2023. **13**(4): p. 483.

29. Wang, Z., et al., *Single-lead ECG based multiscale neural network for obstructive sleep apnea detection.* Internet of Things, 2022. **20**: p. 100613.

30. Wu, H.T., et al., *Phenotype-Based and Self-Learning Inter-Individual Sleep Apnea Screening With a Level IV-Like Monitoring System.* Front Physiol, 2018. **9**: p. 723.

31. Wu, S., et al., *Sleep apnea screening based on Photoplethysmography data from wearable bracelets using an information-based similarity approach.* Computer Methods and Programs in Biomedicine, 2021. **211**: p. 106442.

32. Xu, Y., et al., *Comparative study of a wearable intelligent sleep monitor and polysomnography monitor for the diagnosis of obstructive sleep apnea.* Sleep Breath, 2023. **27**(1): p. 205-212.

33. Yeh, E., et al., *Detection of obstructive sleep apnea using Belun Sleep Platform wearable with neural network-based algorithm and its combined use with STOP-Bang questionnaire.* PLoS One, 2021. **16**(10): p. e0258040.

34. Yeo, M., et al., *Respiratory Event Detection During Sleep Using Electrocardiogram and Respiratory Related Signals: Using Polysomnogram and Patch-Type Wearable Device Data.* IEEE J Biomed Health Inform, 2022. **26**(2): p. 550-560.

35. Yeo, M., et al., *Robust Method for Screening Sleep Apnea With Single-Lead ECG Using Deep Residual Network: Evaluation With Open Database and Patch-Type Wearable Device Data.* IEEE Journal of Biomedical and Health Informatics, 2022. **26**(11): p. 5428-5438.

36. Yüzer, A.H., et al., *A different sleep apnea classification system with neural network based on the acceleration signals.* Applied Acoustics, 2020. **163**: p. 107225.

37. Zhang, H., et al., *Long-Term Sleep Respiratory Monitoring by Dual-Channel Flexible Wearable System and Deep Learning-Aided Analysis.* IEEE Transactions on Instrumentation and Measurement, 2023. **72**: p. 1-9.

38. Zhou, G., et al., *Automatic monitoring of obstructive sleep apnea based on multi-modal signals by phone and smartwatch.* Annu Int Conf IEEE Eng Med Biol Soc, 2023. **2023**: p. 1-4.
